# Phase I Study of Oncolytic Virus VV1 as Monotherapy or in Combination with Avelumab in Patients with Relapsed Refractory Solid Tumors

**DOI:** 10.1158/2767-9764.CRC-25-0803

**Published:** 2026-08-05

**Authors:** Jaime R. Merchan, Manish R. Patel, Timothy P. Cripe, Matthew O. Old, Jen Boughton, Rosa M. Diaz, Kah-Whye Peng, Stephen J. Russell, Luke Russell, Nandakumar Packiriswamy, Erol Wiegert, Alice S. Bexon, Steven F. Powell

**Affiliations:** 1 University of Miami, Miami, Florida.; 2 University of Minnesota, Minneapolis, Minnesota.; 3 Nationwide Children’s Hospital, Columbus, Ohio.; 4 Ohio State University, Columbus, Ohio.; 5 Vyriad, Rochester, Minnesota.; 6 Mayo Clinic, Rochester, Minnesota.; 7 Bexon Clinical Consulting LLC, Montclair, New Jersey.; 8 Sanford Health, Sioux Falls, South Dakota.

## Abstract

**Purpose:**

VV1 is designed to induce selective oncolysis of tumor cells and amplify cellular antitumor immune responses. This open-label, phase I, multicenter clinical trial assessed the safety and tolerability of VV1 monotherapy administered intratumorally or intravenously or administered intravenously in combination with avelumab.

**Patients and Methods:**

Patients with advanced solid tumors received intratumoral (*n* = 27) or intravenous VV1 monotherapy (*n* = 33) or intravenous VV1 plus avelumab (*n* = 16). Infusion durations (15–180 minutes) and viral dose (1.7 × 10^10^ or 1 × 10^11^ TCID_50_) were also evaluated. Study objectives included VV1 safety and tolerability, pharmacokinetics, pharmacodynamics, preliminary efficacy, and immune responses.

**Results:**

Intratumoral and intravenous VV1 were well tolerated, and injection reactions were infrequent. The most common adverse events of any grade related to VV1 were cytokine release syndrome (58%), fatigue (33%), and decreased lymphocyte count (32%). Virus infection of tumors was confirmed by NIS imaging and increases in serum levels of virally encoded interferon-β (IFNβ). Histologic tumor analysis at 28 days after intratumoral VV1 administration indicated increases in tumor-infiltrating immune cells. Two durable partial responses were recorded: a patient with pheochromocytoma after one intravenous dose of VV1 and a patient with thymic cancer in the combination arm. Stable disease was observed in 17 of 49 patients (34.7%) who received intravenous VV1.

**Conclusions:**

This first-in-human study demonstrates that intratumoral or intravenous VV1 administration had an acceptable safety profile as monotherapy and intravenously in combination with avelumab. There is preliminary antitumor activity. Intratumoral VV1 plus cemiplimab is now showing promise in the neoadjuvant setting.

**Significance:**

VV1 is a tumor-selective oncolytic vesicular stomatitis virus engineered to express IFNβ and the thyroidal NIS reporter gene. In this phase I study, we have demonstrated that a single administration of VV1, as a monotherapy or with an immune checkpoint inhibitor is safe, infects tumor lesions resulting in intratumoral infiltration of immune cells, and exhibits early signs of antitumor activity in patients with advanced unresectable and metastatic solid tumors.

## Introduction

In recent years immune checkpoint inhibitors (ICI) have transformed the treatment of solid and hematologic malignancies. Although many patients benefit from ICI-based therapies, a large proportion of tumors remain unresponsive (1). Such immunologically “cold” tumors occur due to a variety of factors, including low burden of tumor neoantigens, defects in dendritic cell antigen processing and migration, failures in interferon (IFN) signaling, and reduced tumor-infiltrating lymphocytes (2). ICI shortcomings have stimulated research into immunotherapies that may (re)sensitize immunosuppressive tumor microenvironments (TME; refs. 3–5).

Oncolytic viruses (OV) selectively infect and promote tumor cell lysis (oncolysis). Oncolysis releases viral progeny that can infect other tumor cells and produce cytokines, damage-associated molecular patterns, pathogen-associated molecular patterns, tumor cell debris, and tumor-associated antigens (3–7). These “danger signals” contribute to activate local and systemic adaptive and innate immune responses, stimulating immune cell recruitment and generating an inflamed, less immunosuppressive TME (3–5).

Talimogene laherparepvec (Imlygic, T-VEC; Amgen, Inc.) for advanced, unresectable melanoma is the first FDA-approved OV (8, 9). Although T-VEC monotherapy demonstrated a survival benefit (10), combination with pembrolizumab did not improve overall or progression-free survival (11). T-VEC is licensed for intratumoral administration which, while facilitating direct delivery and reducing risk of serum inactivation, also poses challenges for metastatic disease and/or difficult-to-access tumors (3, 12–14). In contrast, systemic OV administration has the potential to infect multiple tumor sites (3, 13, 14).

Vesicular stomatitis virus (VSV) is a nonpathogenic negative strand RNA *Rhabdovirus* that normally infects livestock (15, 16). Although VSV has broad tissue tropism, it cannot generate a productive infection in healthy cells due to the type-1 IFN response (7). Cancer cells frequently exhibit IFN signaling defects, making them vulnerable to VSV (7). Furthermore, short replication and a small genome (amenable to genetic manipulation) add to VSV’s attractiveness as a potential anticancer agent (7, 17–19).

VSV–IFNβ–NIS [Voyager-V1 (VV1)] is engineered to encode human IFNβ and the thyroidal sodium iodide symporter NIS (19, 20). IFNβ enhances tumor-selective replication and immune priming in human cancer cells. IFNβ production by infected cancer cells protects noncancer cells from VV1. Consequently, VV1 is cytopathic toward tumor cells, leading to rapid lysis and VV1 amplification (19, 20). NIS expression facilitates ^99m^Tc pertechnetate uptake by infected cells for single photon emission computed tomography (SPECT) or positron emission tomography imaging of viral infection (19, 21). In murine tumor models and dogs with spontaneous tumors, VV1 exhibited potent antitumor activity after one intravenous administration (19, 20). Safety studies in murine models, dogs, and swine support the translation of VV1 into clinical testing (19, 20, 22–24). Furthermore, preclinical data including from a related virus, VSV–IFNβ, combined with ICI blockade, demonstrated significantly enhanced antitumor activity, which is attributed to increased production of tumor antigen–specific T cells (25, 26).

This study involved patients with relapsed or refractory advanced unresectable or metastatic solid tumors for whom alternative treatment options were not appropriate or available. Herein, we describe the safety of intravenous and intratumoral VV1 monotherapy, plus the impact of intravenous infusion duration and dose on tolerability, pharmacokinetics, and pharmacodynamics. We also report upon the safety, tolerability, and preliminary efficacy of intravenous VV1 combined with avelumab (anti–PD-L1).

## Patients and Methods

### Trial design and statistics

**Figure 1.**
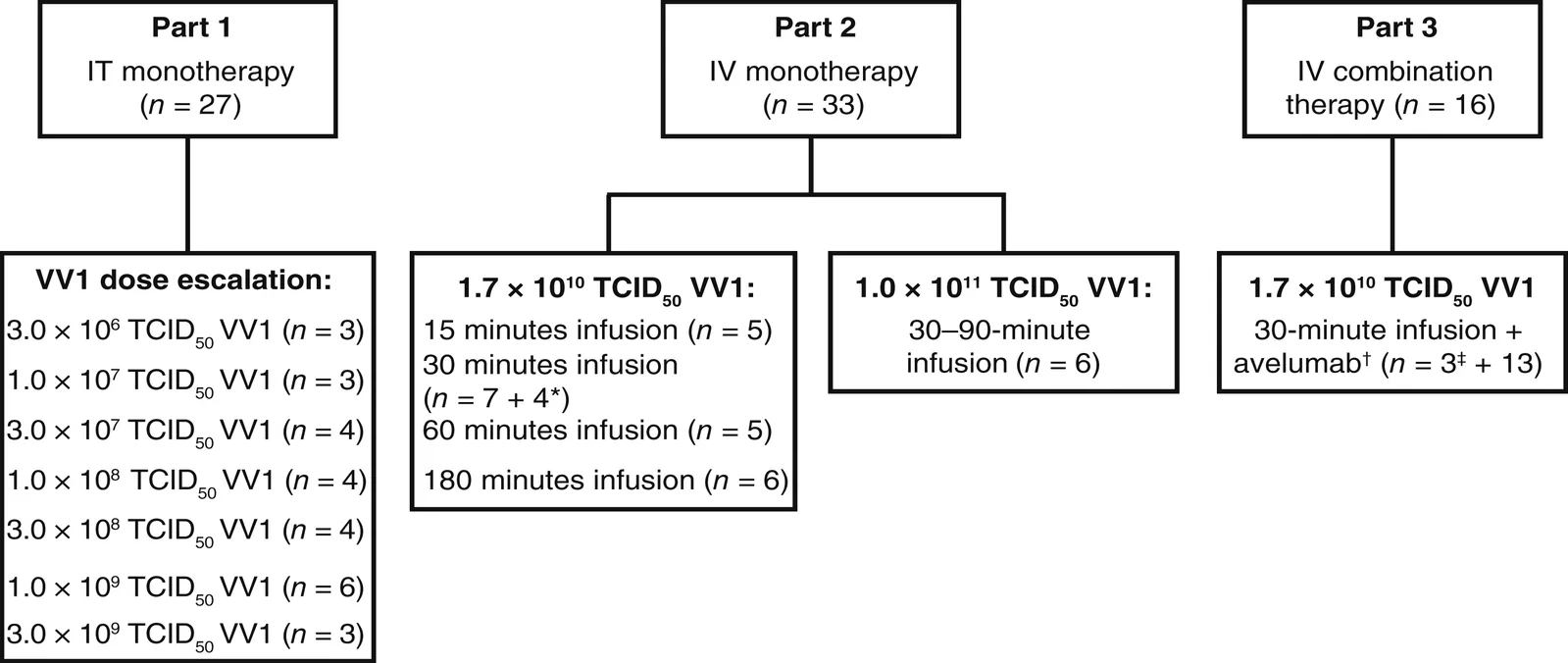
Trial schema for parts 1–3. *Expansion cohort into patients with neuroendocrine tumors (NET). Based on the lack of efficacy in the combination arm, further monotherapy expansion cohorts were abandoned. † Labeled dose/regimen of avelumab. ‡ Combination therapy safety lead in. No attrition was observed.

This was a phase I, multicenter trial (NCT02923466) involving 76 patients, ages 19 to 82 years, comprising three parts (Fig. 1). Patients were assigned sequentially to treatment cohorts by the sponsor after discussion with the principal investigators.

In part 1 (monotherapy intratumoral dose escalation) VV1 was administered into one single target lesion to evaluate safety, preliminary efficacy signals, and mechanism of action. Patients were given doses of VV1 3 × 10^6^, 1 × 10^7^, 3 × 10^7^, 1 × 10^8^, 3 × 10^8^, 1 × 10^9^, and 3 × 10^9^ median tissue culture infectious dose (TCID_50_). In part 2 (monotherapy intravenous dose selection) two dose levels (DL) of intravenous VV1 were investigated: 1.7 × 10^10^ and 1 × 10^11^ TCID_50_ administered over 15, 30, 60, or 180 minutes and 30 to 90 minutes, respectively. Different infusion durations were explored to assess whether a longer or shorter infusion duration mitigated cytokine release syndrome (CRS) symptoms, primarily hypotension. Part 3 was designed to explore the safety and efficacy of 1.7 × 10^10^ TCID_50_ of intravenous VV1 administered over 30 minutes in combination with avelumab.

This study was conducted in compliance with the International Council for Harmonization of Technical Requirements for Pharmaceuticals for Human Use Guideline for Good Clinical Practice as outlined in the current version of 21 CFR, subpart D, part 312, part 50, and part 56, and in general, was consistent with the most recent version of the Declaration of Helsinki.

The appropriate institutional review boards approved the protocol and informed consent form prior to implementation. Voluntary written informed consent was obtained from every patient prior to trial participation.

A statistical analysis plan was written and adhered to, although given the exploratory nature of this study, the sample size was not based upon power calculations. A Simon two-stage design was used for part 3. The null hypothesis was that 3% was the true response rate. This was to be rejected if ≥3 responses occurred in 21 patients. This design yielded a type I error rate of 5% and power of 80% when the true response rate was 20%.

Primary objectives were to assess the safety and tolerability of intravenous and intratumoral VV1 monotherapy and intravenous VV1 combined with avelumab. Secondary objectives were to evaluate preliminary antitumor activity and response rates, pharmacokinetic and pharmacodynamic profiles, and biodistribution of VV1 monotherapy and in combination with avelumab.

### Study drugs and administration

VV1 is a live virus engineered by inserting the human IFNβ gene downstream of the M gene and the NIS gene (cDNA) downstream of the G-protein gene into a molecular clone of an Indiana strain VSV (19, 27). VV1 was manufactured in accordance with Good Manufacturing Practice guidelines and stored at < −65°C until use. VV1 was considered a “biological substance, category B” based on lack of transmissibility in host species in compliance with International Air Transportation Association and Department of Transportation regulations (24). VV1 was thawed and diluted immediately prior to administration (30 minutes to 6 hours). For intratumoral administration, VV1 was diluted in normal saline (NS) with 1% human serum albumin (HSA): volume was dependent on the size of the lesion to be injected (Supplementary Table S1). For intravenous administration, VV1 was diluted in 100 mL of NS with 1% HSA.

We designed a premedication strategy to mitigate VV1 infusion-related reactions (IRR), which evolved throughout the study. The final recommendation, prior to VV1 infusion, was 1L NS, diphenhydramine (25–50 mg), acetaminophen (325–650 mg), naproxen 500 mg [or equivalent nonsteroidal anti-inflammatory drug (NSAID)], and antinausea medications (prochlorperazine 10 mg and ondansetron 8 mg).

Avelumab was prepared and administered (every 2 weeks) as per the package insert. To mitigate avelumab-related IRRs, patients were premedicated with diphenhydramine (25–50 mg) and acetaminophen (325–650 mg) 30 to 60 minutes prior to the first four avelumab infusions. Premedication for subsequent avelumab infusions was based upon clinical judgment and presence/severity of prior reactions.

### Patient inclusion and exclusion criteria

The patient population (parts 1–3: Fig. 1) included subjects with histologically confirmed diagnosis of an advanced and/or metastatic solid tumor that had relapsed and/or was refractory to standard therapy, requiring progression on at least one prior line of therapy in the relapsed/metastatic setting and with no existing options able to provide clinical benefit. Patients had measurable disease based on Response Evaluation Criteria in Solid Tumors 1.1 (RECIST 1.1). At least one patient per cohort in part 1 had ≥2 measurable target lesions: one for injection and one for noninjected control (Supplementary Table S1). In part 3, patients had either a tumor type known to be refractory to ICI or their disease had progressed on (or following) treatment with an anti–PD-(L)1 antibody. Patients were excluded if they had high volume disease (assessed clinically and radiologically); immune deficiency or bone marrow transplant in the last 5 years; active primary or metastatic central nervous system malignancy; or had been receiving radiotherapy, chemotherapy, or molecularly targeted agents or tyrosine kinase inhibitors within 2 weeks or 5 half-lives (whichever is longer) of the start of study treatment; immunotherapy/monoclonal antibodies within 3 weeks of the start of study treatment; nitrosoureas; antibody–drug conjugates; or radioactive isotopes within 6 weeks of the start of study treatment.

### Safety assessments

Safety was assessed using the National Cancer Institute Common Terminology Criteria for Adverse Events (CTCAE) version 4.03 and Lee and colleagues (28) for CRS grading criteria. Safety assessments included adverse events (AE), serious AEs (SAE), physical examinations, vital sign measurements, Eastern Cooperative Oncology Group status, clinical safety laboratory evaluations (hematology, serum chemistry, coagulation, T4/TSH, urinalysis, and hepatic function), and electrocardiogram.

Dose-limiting toxicity (DLT) was defined as an AE attributed (definitely, probably, or possibly) to VV1 (and/or avelumab), occurring during the DLT evaluation period, and meeting the criteria outlined in Supplementary Tables S2 and S3.

Dropouts were closely monitored and could be replaced during escalation.

### IRR and CRS management

Due to CRS events in the intravenous monotherapy cohort and IRR risk in the avelumab combination cohort, IRR/CRS management guidelines were developed. Due to hypotension events, on the day of VV1 administration, holding antihypertensive medications (no more than 1 medication) for patients with well-controlled hypertension were advised per the investigator. Premedication options, previously described, were administered to patients, based on the investigator’s decision. Readministration of these medications after the infusion was per decision of the investigator. Approximately 4 hours after the start of VV1 infusion and/or immediately at the start of any CRS/IRR symptoms, repeat treatment with the same doses of diphenhydramine, acetaminophen, and naproxen/other NSAID was recommended. Additional supportive care, including cooling or warming devices and meperidine, were allowed for participants experiencing breakthrough fevers and/or rigors. Additional CRS management for grade ≥2 CRS was adapted per Lee and colleagues 2014. CRS events observed in this study were self-limited and well managed with supportive measures. The time on event for CRS did not warrant adding tocilizumab to the recommended measures.

### Response assessment

Tumor assessment followed RECIST 1.1 using CT (preferred) or magnetic resonance imaging. Imaging was performed at baseline (≤28 days prior to treatment) and day 43 (±2 days) and then every 6 weeks until progressive disease (PD). Responses were confirmed per RECIST 1.1, and tumor size change was calculated for individual lesions.

### Pharmacokinetic and pharmacodynamic analyses

Quantitative reverse transcriptase polymerase chain reaction (qRT-PCR) was used to determine VSV-nucleocapsid (N) RNA copies in whole blood (viremia, representing systemic exposure for pharmacokinetic purposes; ref. 23). Whole blood was collected into Paxgene RNA tubes at pretreatment, end of infusion, and 4 hours and 1, 2, 7, 14, and 21 days after treatment. Pharmacodynamic assessments included expression of virally encoded NIS via SPECT/CT and analysis of serum IFNβ by enzyme-linked immunosorbent assay (PBL, cat. #41415-1). To assess viral shedding, urine and buccal samples were collected on days 1 (pre-treatment) 8, 15, 22 and 43 and analyzed using PCR or by overlay on Vero cells to detect infectious virus. If a patient’s specimen was negative on day 8, or any day thereafter, no further samples were collected.

### Cytokine expression

Cytokines at baseline and 4 hours after VV1 infusion were measured using a multiplex cytokine kit (Thermo Fisher Scientific, ProcartaPlex Human inflammation panel, cat. #EPX200-12185-901). Anti-VV1 neutralizing antibodies (NAb) were measured at baseline and day 28 using standard virus plaque reduction neutralization assays with VSV-GFP as the indicator virus [methodology available upon request (29)].

### Mechanism of action of VV1 *in situ*

SPECT/CT was used to assess VV1 tumor infection and viral replication in at least one patient per DL in part 1. SPECT/CT was performed after intravenous ^99m^Tc pertechnetate administration (45 minutes after 20 mCi) at baseline and 2 days after treatment and on additional days if the day 2 scan was NIS-positive. The presence/absence of NIS-positive tumors was read by the investigator and a central radiologist.

In part 1, to better understand the mechanism of action of VV1, when feasible, both injected and noninjected metastatic tumors were biopsied before (day −7 to 0) and after VV1 administration (day 28 ± 3 days), and the histology results were compared.

### Histologic analyses

Immunohistochemistry (IHC) was used to assess T-cell infiltration in paraffin embedded and sectioned (5 μm) tumor biopsies from patients in part 1. Tumor biopsies were obtained within 28 days before enrollment. Injected and noninjected tumors that were NIS-positive on SPECT/CT or still present were biopsied (if feasible) between 8 and 22 days following VV1 injection. Slides were subjected to antigen retrieval, stained using antibodies for CD3 (Dako, F7.2.38), CD4 (Dako. 4B12), CD8 (Dako, 144B), PD-1 (Abcam, NAT105), PD-L1 (Dako, 22C3), and CD68 (Dako, PG-M1) and detected with a secondary antibody using ultraView Universal DAB Detection Kit (Ventana). Images were acquired using the Hamamatsu NanoZoomer XR scanner.

## Results

### Patients

Between April 2017 and June 2021, 76 patients with advanced refractory or unresectable solid tumors, with a majority of colorectal cancers and a few rarer histologies, received VV1 as monotherapy or in combination with avelumab (Fig. 1). All patients were evaluable for safety and efficacy.

In part 1, 27 patients across 7 escalating dose cohorts received intratumoral VV1 into one target lesion at doses ranging from 3 × 10^6^ to 3 × 10^9^ TCID_50_ per patient. The median patient age was 61 years, with a median of four prior lines of treatment (range, 1–9). Thirteen patients (48%) were female (Table 1; Supplementary Table S4).

**Table 1.** Baseline demographics.

​	Part 1	Part 2	Part 3
	(*N* = 27)	(*N* = 33)	(*N* = 16)
Age (years)			
Median (min–max)	61 (36–79)	61 (19–82)	60 (45–68)
Sex, *n* (%)			
Male	14 (51.9)	11 (33.3)	11 (68.8)
Female	13 (48.1)	22 (66.7)	5 (31.3)
Race			
Black or African American	3 (11.1)	3 (9.1)	1 (6.3)
White	22 (81.5)	30 (90.9)	13 (81.3)
Other/not reported	2 (7.4)	0 (0)	2 (12.5)
Ethnicity			
Hispanic or Latino	3 (11.1)	5 (15.2)	2 (12.5)
Non-Hispanic or Latino	24 (88.9)	28 (84.8)	13 (81.3)
Unwilling to provide	0	0	1 (6.3)
Prior anticancer regimens			
Median (min, max)	4 (1–9)	3 (1–12)	3 (2–12)
ECOG, *n* (%)			
Grade 0	8 (29.6)	19 (57.6)	8 (50)
Grade 1	19 (70.4)	14 (42.4)	8 (50)
Child–Pugh class, *n* (%)			
A (5–6 points)	26 (96.3)	33 (100)	14 (87.5)
B (7–9 points)	0 (0)	0 (0)	2 (12.5)
Missing	1 (3.7)	0 (0)	(0)
Primary tumor, *n* (%)			
Colorectal adenocarcinoma	7 (25.9)	13 (39.4)	13 (81.3)
NET	0 (0)	7 (21.2)^a^	0 (0)
Head and neck/esophagus SCC^b^	5 (18.5)	1 (3)	0 (0)
Breast adenocarcinoma	2 (7.4)	1 (3)	1 (6.3)
Pancreatic adenocarcinoma	3 (11.1)	1 (3)	0 (0)
Sarcoma	0 (0)	3 (9.1)^c^	0 (0)
Pheochromocytoma	0 (0)	2 (6.1)	0 (0)
Prostate adenocarcinoma	0 (0)	1 (3)	1 (6.3)
Serous ovarian	1 (3.7)	1 (3)^d^	0 (0)
Carcinoma unknown primary	1 (3.7)	1 (3)	0 (0)
Other	8 (29.6)^e^	2 (6.1)^f^	1 (6.3)^g^

Abbreviations: ECOG, Eastern Cooperative Oncology Group; NET, neuroendocrine tumor; SCC, squamous cell carcinoma.

a 5 lung, 1 pelvic bones, and 1 rectal.

b 1 esophageal in part 1, others head and neck.

c 1 myofibroblastic skeletal muscle and 2 uterine: leiomyosarcoma and endometrial stromal.

d 1 mixed endometrioid/serous.

e 1 of each: adenoid cystic carcinoma of head and neck, pleural mesothelioma, cervix adenocarcinoma, clear cell endometrial carcinoma, hard palate adenocarcinoma, ocular melanoma, non–small cell lung cancer, renal cell carcinoma.

f 1 of each: squamous cell carcinoma of anus and adrenal cortical adenocarcinoma.

g 1 thymic carcinoma.

In part 2, 33 patients received a single intravenous infusion of VV1. Twenty-three patients were administered VV1 1.7 × 10^10^ TCID_50_ (*n* = 23) across four cohorts of differing infusion duration (15–180 minutes). Four patients with NET received VV1 1.7 × 10^10^ TCID_50_ administered over 30 minutes. A further cohort of six patients received “high-dose” VV1 1 × 10^11^ TCID_50_ administered over 30 to 90 minutes. Eleven patients (33%) were male. The median patient age was 61 years (range, 19–82) with a median of three prior lines of systemic anticancer therapy (range, 1–12; Table 1; Supplementary Table S5).

Sixteen patients were enrolled in part 3, of whom 13 had microsatellite-stable metastatic castration-resistant colorectal cancer and 3 had other solid tumors (Table 1; Supplementary Table S6). Patients in part 3 had received a median of three prior lines of systemic anticancer therapy (range, 2–12), the median age was 60 years, and 5 (31%) were female. A total of 16 patients received a single VV1 infusion at a dose of 1.7 × 10^10^ TCID_50_ plus avelumab 800 mg intravenously 3 hours later.

### Safety

The majority of treatment-related AEs, irrespective of administration route, occurred within 48 hours of VV1 administration and were short-lived, manageable, and related to viremia and associated cytokine increases; with the exception of a few minor laboratory abnormalities, all resolved within 8 days.

Overall, in 76 enrolled patients, the most common (>15%) VV1-related treatment-emergent AEs (TEAE; CTCAE v4.03) of any grade were CRS (58%), fatigue (33%), decreased lymphocyte count (32%), nausea (29%), pyrexia (20%), and decreased appetite (18%). Forty-three (57%) patients experienced grade 3 to 4 VV1-related TEAEs, the most common (>10%) being decreased lymphocyte count (30%) and lymphopenia (13%).

**Figure 2.**
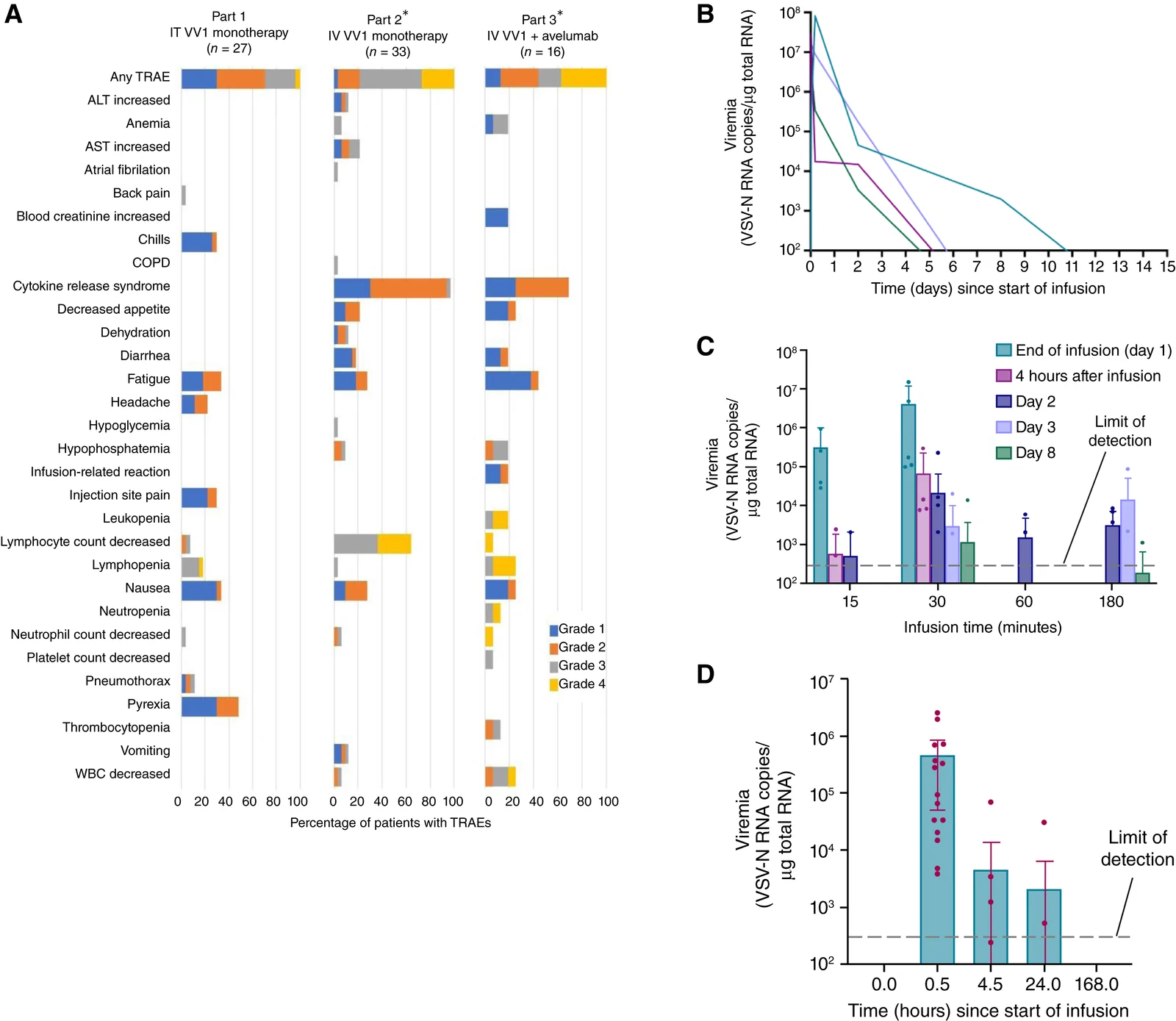
TRAEs and viremia following VV1 monotherapy and in combination with avelumab. **A,** All TRAEs observed in ≥15% of patients by grade, plus all treatment-related grade 3 and 4 events. *After part 1, CRS type symptoms were documented with checkbox in the CRF. **B,** Viremia following 30–90-minute intravenous (IV) infusions of high-dose VV1 (1 × 10^11^ TCID_50_) monotherapy (part 2). Each line represents a unique patient (*n* = 4). **C,** Viremia following 15–180-minute IV infusions of VV1 (1.7 × 10^10^ TCID_50_) monotherapy (part 2). **D,** Viremia following 30-minute IV infusions of VV1 (1.7 × 10^10^ TCID_50_) + labeled dose/regimen of avelumab (part 3). ALT, alanine aminotransferase; COPD, chronic obstructive pulmonary disease; TRAE, treatment-related AE.

As of January 21, 2021, intratumoral monotherapy dose escalation (part 1) was completed, and no DLTs were identified up to doses of 3 × 10^9^ TCID_50_. Among patients administered intratumoral VV1, the most commonly reported (>15% patients) VV1-related TEAEs were pyrexia (48%), fatigue (33%), nausea (33%), chills (30%), injection site pain (30%), headache (22%), and lymphopenia (19%). The vast majority of TEAEs were mild-to-moderate. VV1-related grade 3 to 4 TEAEs were observed in 8 of 27 patients; these were lymphopenia (19%), decreased neutrophil count (4%), pneumothorax (4%), decreased lymphocyte count (4%), and back pain (4%; Fig. 2A).

The most common (>15%) TEAEs related to VV1 intravenous monotherapy (part 2) was CRS (97%). The majority of CRS events were grade 1 (30%) and grade 2 (64%); 3% of patients experienced CRS grade 3; there were no grade 4 events (Fig. 2A). Other common VV1-related TEAEs were lymphocyte count decrease (64%), nausea (27%), fatigue (27%), decreased appetite (21%), increased aspartate aminotransferase [AST (21%)], and diarrhea (18%). VV1-related grade 3 to 4 TEAEs occurred in 26 of 33 patients, with the most common (>5%) being transient decreased lymphocyte count (64%), anemia (6%), and increased AST (9%; Fig. 2A).

No withdrawals or deaths related to VV1 were observed in part 2. Seven VV1-related SAEs, as per Lee and colleagues criteria (28), were reported: all were CRS grade 2, except with the 15–minute infusion where there was one grade 3 CRS (also a DLT; Table 2), which was characterized by hypoxia and was treated with oxygen applied via a Venturi mask. Following the risk profile of the drug, the episode was short-lived (overnight) and self-limited. In the 1.0 × 10^11^ TCID_50_ cohort, a grade 3 SAE (also a DLT) of atrial fibrillation was reported. The 15-minute infusion cohort was discontinued because of observation of a greater number of infusion-related AEs, mainly CRS but also a DLT of grade 2 hyperbilirubinemia. The incidence of AEs observed among patients treated with the higher dose of intravenous 1 × 10^11^ TCID_50_ over 30 to 90 minutes were similar to those reported among patients given 1.7 × 10^10^ TCID_50_ intravenously over 30 minutes_._ Two intravenous cohort patients received tocilizumab with no evidence of benefit given the self-limited nature of CRS. Therefore, based on these observations, the 30-minute 1.7 × 10^10^ TCID_50_ intravenous infusion was chosen as the dosing regimen for part 3 and future studies.

**Table 2.** IRRs and CRS related to intravenous VV1 monotherapy (part 2).

Cohort (*n*)	VV1 intravenous dose (TCID_50_)	CRS max Grade 1 *N* (%)	CRS max Grade 2 *N* (%)	CRS max Grade 3 *N* (%)
VV1 15 minutes (5)	1.7 × 10^10^	1 (20)	3 (60)	1^a^ (20)
VV1 30 minutes (7)	1.7 × 10^10^	1 (14.3)	6 (85.7)	—
VV1 60 minutes (5)	1.7 × 10^10^	3 (60)	2 (40)	—
VV1 180 minutes (6)	1.7 × 10^10^	2 (33.3)	4 (66.7)	—
VV1 30–90 minutes (6)	1 × 10^11^	1 (16.7)	4 (66.7)	—
VV1 30 minutes (4)^b^	1.7 × 10^10^	2 (50)^c^	2 (50)	—
Total		10 (30.3)	21 (63.6)	1 (3)

a One grade 3 was considered a DLT.

b Expansion cohort into patients with NET.

c Recorded as IRR.

There was no evidence of a difference in the risk profile of VV1 when it was combined with avelumab. The most common (>15%) TEAEs related to intravenous VV1, with avelumab (part 3), were CRS (69%), fatigue (44%), nausea (25%), decreased appetite (25%), reduced white blood cell (WBC) count (25%), lymphopenia (25%), IRR (19%), diarrhea (19%), and hypophosphatemia (19%). VV1-related grade 3 to 4 TEAEs occurred in 9 of 16 patients, with the most common (≥10%) being lymphopenia (25%), reduced WBC count (19%), leukopenia (19%), anemia (13%), hypophosphatemia (13%), and neutropenia (13%; Fig. 2A).

### Antitumor activity

#### Intratumoral VV1 monotherapy

Of 27 patients, 23 were assessed as per RECIST 1.1: 9 with stable disease (SD) and 14 with PD at day 43 (Table 3). There were 15 tumor lesions (6 injected and 9 control) that exhibited measurable reductions in length. Five tumor lesions (3 injected and 2 control) showed no change. The maximum decrease in tumor lesion length of 21.9% was observed in a control lesion, in a patient with endometrial clear cell carcinoma who had received VV1 1 × 10^9^ TCID_50_ intratumorally.

**Table 3.** Best overall response by RECIST 1.1 for VV1 monotherapy and in combination with avelumab (parts 1–3).

Cohort (*n*)	VV1 intravenous dose (TCID_50_)	Patients with assessed disease *n* (%)	CR	PR	SD	PD	N/A
Part 1^a^							
DL1 (3)	3 × 10^6^	3 (100)	0	0	0	3 (100)	0
DL2 (3)	1 × 10^7^	1 (33.3)	0	0	0	1 (33.3)	2 (66.7)
DL3 (4)	3 × 10^7^	4 (100)	0	0	1 (25)	3 (75)	0
DL4 (4)	1 × 10^8^	4 (100)	0	0	3 (75)	1 (25)	0
DL5 (4)	3 × 10^8^	3 (75)	0	0	1 (25)	2 (50)	1 (25)
DL6 (6)	1 × 10^9^	5 (83.3)	0	0	4 (66.7)	1 (16.7)	1 (16.7)
DL7 (3)	3 × 10^9^	3 (100)	0	0	0	3 (100)	0
All (27)	—	23 (85.2)	0	0	9 (33.3)	14 (51.9)	4 (14.8)
Part 2^b^							
VV1 15 minutes (5)	1.7 × 10^10^	4 (80)	0	0	2 (40)	2 (40)	1 (20)
VV1 30 minutes (7)	1.7 × 10^10^	7 (100)	0	1 (14.3)^c^	3 (42.9)	3 (42.9)	0
VV1 60 minutes (5)	1.7 × 10^10^	5 (100)	0	0	2 (40)	3 (60)	0
VV1 180 minutes (6)	1.7 × 10^10^	6 (100)	0	0	1 (16.7)	5 (83.3)	0
VV1 30–90 minutes (6)^d^	1 × 10^11^	6 (100)	0	0	1 (16.7)	5 (83.3)	0
VV1 30 minutes (4)^e^	1.7 × 10^10^	4 (100)	​	​	2 (50)	2 (50)	0
All (33)	—	32 (97)	0	1 (3)	11 (33.3)	20 (60.6)	1 (3)
Part 3^b^							
Safety lead in VV1 + avelumab (3)	1.7 × 10^10^	3 (100)	0	1 (33.3)^f^	2 (66.7)	0	0
VV1 + avelumab mCRC (13)	1.7 × 10^10^	12 (92.3)	0	0	4 (30.8)	8 (61.5)	1 (7.7)
All (16)	—	15 (93.8)	0	1 (6.3)	6 (37.5)	8 (50)	1 (6.3)

Abbreviations: CR, complete response; N/A, not assessed; TCID_50_, tissue culture infectious dose required to infect 50% of a culture.

**Figure 3.**
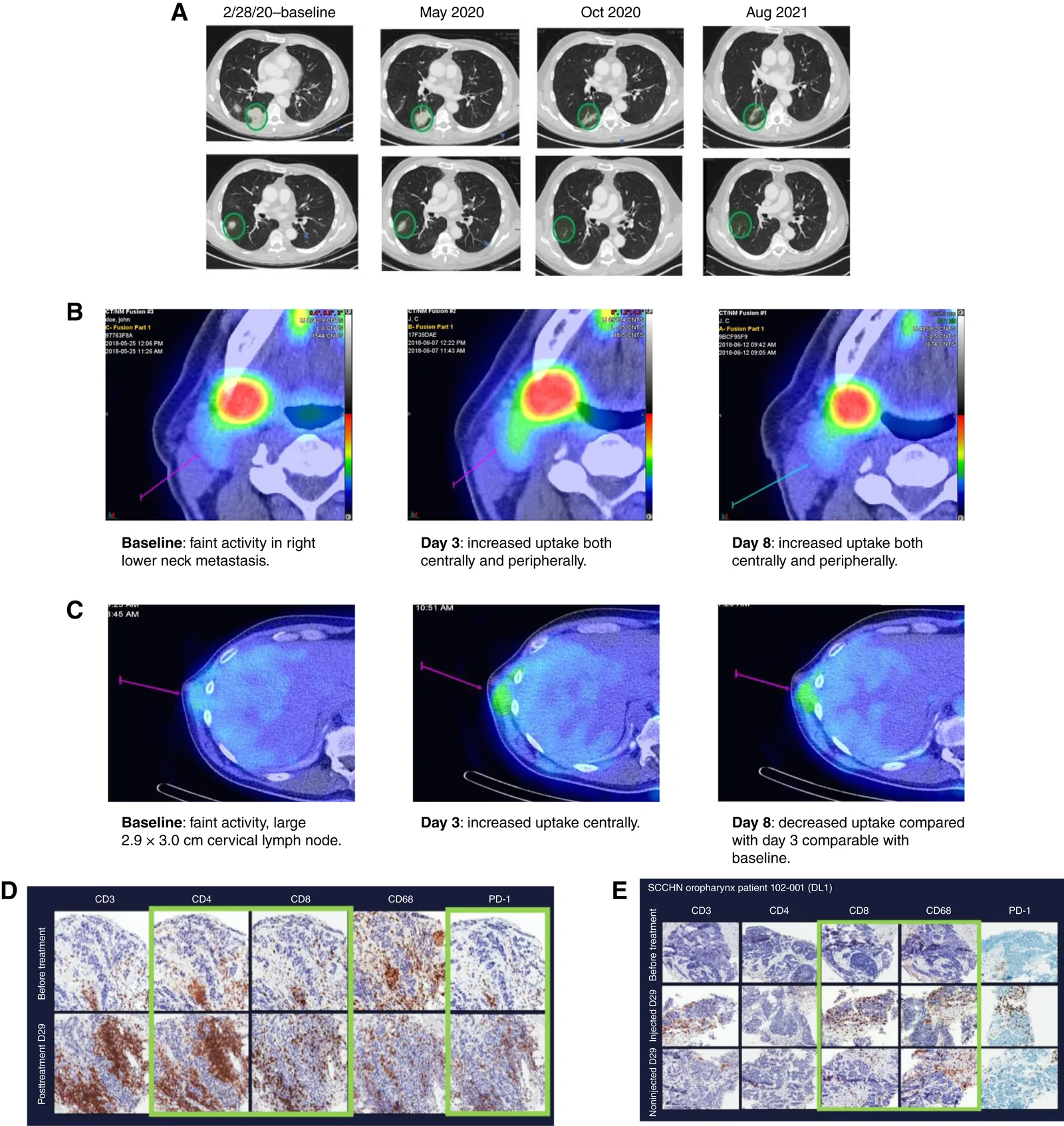
Imaging following intratumoral (IT) and intravenous (IV) VV1. **A,** CT images of the thoracic region of a 57-year-old patient with thymic carcinoma following a 30-minute intravenous infusion of VV1 (1.7 × 10^10^ TCID_50_) + labeled dose/regimen of avelumab (part 3). In the opinion of the investigator, only scar tissue remained at the last assessment (August 2021). **B,** SPECT/CT/NIS imaging of 75-year-old male patient with adenocarcinoma (unknown primary cancer) following intratumoral (IT) administration of VV1 (3 × 10^8^ TCID_50_) monotherapy (part 1). **C,** SPECT/CT/NIS imaging of a 62-year-old male patient with mesothelioma following IT administration of VV1 (1 × 10^8^ TCID_50_) monotherapy (part 1). **D,** Immune cell infiltrates and PD-1 in a male patient with head and neck squamous cell (HNSCC) with SD, following IT administration of VV1 (part 1). Images are of an injected lesion. **E,** Immune cell infiltrates and PD-1 in a 70-year-old male patient with HNSCC with SD, following IT administration of VV1 (3 × 10^6^ TCID_50_) monotherapy (part 1). Top, baseline. Middle, injected lesion. Bottom, noninjected lesion.

a Responses based on investigator evaluation at day 43 and not confirmed with a second evaluation.

b Responses based on best single investigator evaluation and not confirmed with a second evaluation.

c Central read hepatic cyst as PD. The investigator reviewed the scan and categorized the patient as having a PR.

d All patients received a 30-minute infusion.

e Expansion cohort into patients with NET.

f Confirmed as a CR in follow-up, see antitumor activity section and Fig. 3A.

#### Intravenous VV1 monotherapy

In part 2, 11 of 33 patients had SD and 20 had PD (Table 3). The best objective response in part 2 was a technically unconfirmed partial response (PR) which nevertheless led to long-term clinical benefit in a patient with pheochromocytoma. This was a 28-year-old female patient, diagnosed in 2005 with stage intravenous pheochromocytoma with metastases to liver, lung, pelvic bones, vertebral column, lower extremities, and multiple lymph nodes. She had received three prior lines of chemotherapy (including ICIs), radiotherapy to the femur, vertebral column, and right abdominal cavity with tumor resection, right femur open reduction, and internal fixation with rodding. In February 2019, the patient received one dose of VV1 at 1.7 × 10^10^ TCID_50_ infused over 30 minutes. Two weeks later, she reported a marked decrease in pain symptoms and was able to reduce and subsequently discontinue opioids. In March 2019, disease evaluation was RECIST PR with 75% reduction in the target lesion. Per protocol, no further scans were performed. At the last contact, in 2022, the patient was alive and doing well with no further systemic treatment.

#### Combination: VV1 plus avelumab

Of the 16 evaluated patients in part 3, six patients had SD and eight had PD (Table 3). There was one patient with a confirmed PR. This was a 57-year-old male patient who was diagnosed with medullary thymic carcinoma in November 2017. Prior to receiving VV1 plus avelumab, he had four lines of systemic therapy. In July 2018, the disease had spread to the liver, ribs, and supraclavicular lymph nodes. His metastases responded to pembrolizumab and cabozantinib. In June 2019, he presented with new lung metastases prior to study start. In March 2020, he received VV1 and started avelumab. At week 6, he experienced a RECIST PR with 68% reduction in the size of the target lesion over baseline (Fig. 3A). At the study end, the patient had no evidence of disease (not biopsy proven). The patient discontinued study participation in January 2022, while continuing to receive compassionate use avelumab. In early 2023, the patient had a local recurrence in his inguinal lymph node. This was treated with radiotherapy while continuing avelumab. Since then, the patient has remained relapse-free and, as of February 2025, was off therapy.

### Correlative studies

#### VV1 pharmacokinetics

The pharmacokinetics of VV1 in the blood (viremia) was monitored over time by analyzing VSV-N RNA in whole blood (VSV-N copies per μg RNA). In part 1, viremia was below the limit of detection (<300 copies per μg) for all patients except one patient who had 4.96 × 10^4^ VSV-N copies per μg RNA 2 days after intratumoral VV1 administration (Supplementary Table S7). However, by day 3, VSV-N had dropped below the limit of detection.

In patients given intravenous VV1, viremia was highest at the end of infusion and declined over time (Fig. 2B–D). There was no secondary viremia observed. Patients given 1 × 10^11^ VV1 tended to have higher viremia at the end of infusion (1.2 × 10^7^ ± 6.3 × 10^6^ copies VSV-N/μg RNA, *n* = 4) after infusion compared with patients who received 1.7 × 10^10^ VV1 (3.94 × 10^6^ ± 2.81 × 10^6^ copies/μg RNA, *n* = 5, 30 minutes infusion group), but the values were not statistically significant (*P* = 0.12, *t* test). Viremia was below the limit of detection in all patients in part 3 by day 8. In part 2, viremia was below the limit of detection in all patients but one (1.2 × 10^3^ copies per μg) by day 15.

Evaluation of VSV-N RNA from buccal samples, by qRT-PCR, indicated that shedding was minimal, with VSV-N RNA below the limit of detection in all but one patient (parts 1, 2, and 3) and at all time points (Supplementary Table S8). No infectious virus was recovered. Urine samples for shedding analyses were only collected from patients in part 1, and all samples were below the limit of detection.

#### VV1 pharmacodynamics

**Figure 4.**
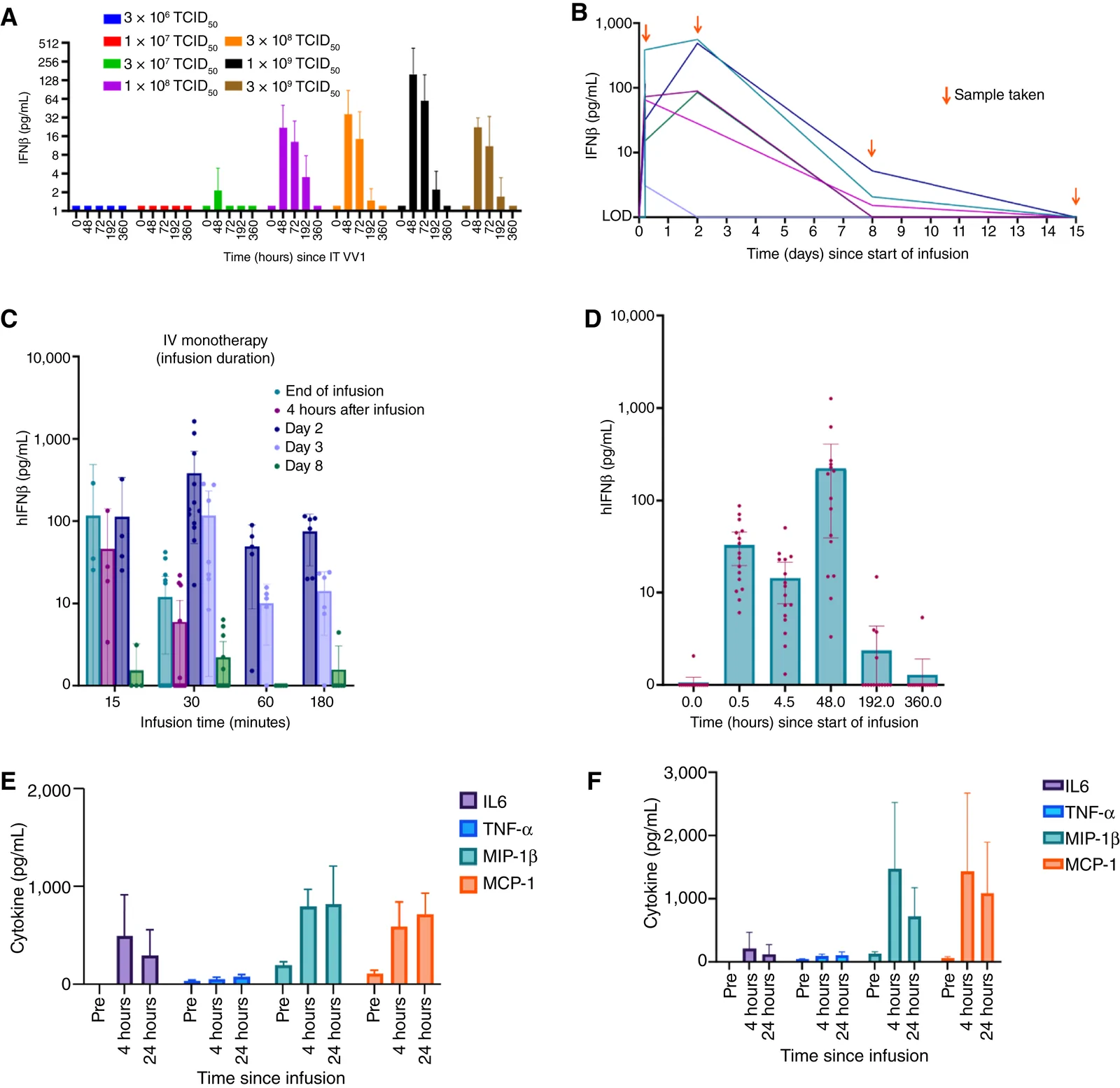
Serum IFNß and cytokine levels following VV1 monotherapy and in combination with avelumab. **A,** Mean serum IFNß following IT administration of VV1 monotherapy dose escalation (part 1). Each DL has 3–6 patients. **B,** Serum IFNβ following 30–90-minute IV infusions of high-dose VV1 (1 × 10^11^ TCID_50_) monotherapy (part 2). Each line represents a unique patient (*n* = 6). **C,** Mean [95% confidence interval (CI)] serum IFNβ following IV infusions of VV1 (1.7 × 10^10^ TCID_50_) monotherapy (part 2). Each dot represents a unique patient (*n* = 23). Limit of detection for IFNβ was 1.2 pg/mL. **D,** Mean (95% CI) serum IFNβ over time following 30-minute IV infusions of VV1 (1.7 × 10^10^ TCID_50_) + labeled dose/regimen of avelumab (part 3). Each dot represents a unique patient (*n* = 16). Limit of detection for IFNβ was 1.2 pg/mL. **E,** Mean (±SEM) cytokine levels following 30-minute IV infusion of VV1 (1.7 × 10^10^ TCID_50_) monotherapy (part 2; *n* = 7). **F,** Mean (±SEM) cytokine levels following 30-minute IV infusion of high dose VV1 (1 × 10^11^ TCID_50_) monotherapy (part 2; *n* = 6).

Virally encoded serum IFNβ, a biomarker of VV1 infection, was highest at 1 to 2 days after administration and declined thereafter (Fig. 4A–D). As expected, IFNβ levels were generally lower following intratumoral administration compared with intravenous administration (Fig. 4A and B). With intratumoral administration, there was a trend for increasing IFNβ expression with increase in dose, peaking at 1 × 10^9^ TCID_50_, suggesting there is a threshold VV1 dose required to establish virus infection (Fig. 4A). In part 2, peak IFNβ serum was highest following a 30-minute infusion (*n* = 7) compared with ≥1-hour infusions (*n* = 11) *P* = 0.0145 (Fig. 4C). The majority of all patients showed peak IFNβ on day 1 or 2 and declined thereafter.

A multiplex cytokine assay was used to measure the acute changes in endogenous cytokines at baseline and 4 and 24 hours after VV1 infusion. There was transient elevation in IL-6, MIP-1β, and MCP-1 at 4 hours after infusion. These results indicate an early immune activation and inflammatory response to VV1 infusion, with cytokines generally declining thereafter (Fig. 4E and F).

Patients mounted a robust humoral antiviral response to VV1 (Supplementary Table S9). In all patients in part 1 and three patients in part 2, anti-VV1 NAb titers were monitored by determining the plaque reduction virus neutralizing titer, adapted from a previously published method (29).

### *In vivo* virus tracking

SPECT/CT showed positive VV1 infection, resulting in NIS expression in tumor cells and uptake of ^99m^Tc pertechnetate in patients from part 1 (Fig. 3B and C). Of 23 evaluable patients, 11 were confirmed as NIS-positive by investigator read, compared with 7 by central read. Uptake of ^99m^Tc pertechnetate by NIS-expressing cells was highest at day 3 and declined thereafter, indicating infected cell death and termination of the viral infection by day 8.

### IHC staining

Biopsies were taken from lesions positive for SPECT/CT or still present between 8 and 22 days after intratumoral VV1 administration. Tumor sections were stained with antibodies for CD3, CD4, CD8, CD68, PD-1, and PD-L1. The density of antigen-positive cells in the target lesions and nontarget lesions collected at baseline, before treatment, and day 28 after intratumoral VV1 was photographed. Representative images are shown in Fig. 3D and E. In some tumor biopsies, there was increased infiltration of immune cells CD4, CD8, and CD68 after intratumoral VV1 compared with before treatment.

## Discussion

This study demonstrates that intratumoral and intravenous VV1 monotherapy and intravenous VV1 in combination with avelumab have a good tolerability profile and exhibit preliminary signs of antitumor activity in patients with advanced solid tumors.

Intratumoral and intravenous administration of VV1 were both well tolerated by patients. In part 1, no DLTs were observed up to doses of 3 × 10^9^ TCID_50_. In addition, in part 2, no DLTs occurred in 18 patients receiving intravenous VV1 monotherapy at a dose of 1.7 × 10^10^ TCID_50_ over 30 minutes (7 patients), 60 minutes (5 patients), and 180 minutes (6 patients). The safety profile between the three longer dosing cohorts (30–180 minutes) was comparable. However, there were two DLTs in the five patients administered VV1 via a 15-minute infusion: one case of transient grade 3 hypoxia that recovered within 1 day, and one case of grade 2 hyperbilirubinemia associated with grade 3 increase in AST. Therefore, we decided that infusion durations below 30 minutes would not be further explored. IRRs/CRS were manageable with overnight hospitalization and supportive care. The short time on event limits the indication for the use of tocilizumab. No infectious virus was detected in the urine or buccal cavity at any time point, indicating that there is little or no risk to carers and no environmental impact. There was no secondary viremia and no significant difference in viremia between DLs, although patients given intravenous 1 × 10^11^ TCID_50_ VV1 tended to have higher viremia at 4 hours after infusion, which might favor better tumor penetration at the highest dose.

In summary VV1 1.7 × 10^10^ TCID_50_ intravenously over 30 minutes in combination with avelumab, 1 × 10^11^ TCID_50_ intravenously over 30 minutes in monotherapy, and 3 × 10^9^ TCID_50_ intratumorally were selected for phase II studies.

The response variability observed with systemic OVs may be due to a number of factors, including host antiviral immunity, dose dilution, tumor cell susceptibility to viral replication, and permeability of tumor microvessels to the circulating virus (13, 30, 31). This study indicates that infusion duration may affect clinical response. After receiving intravenous VV1 monotherapy, a 28-year-old female, previously treated with three lines of chemotherapy for advanced pheochromocytoma, achieved clinical benefit lasting 28 months (unconfirmed PR), and a 57-year-old male patient, previously treated with four lines of systemic therapy and multiple rounds of radiotherapy for medullary thymic carcinoma, achieved a confirmed PR after lasting more than 2 years, following treatment with VV1 plus avelumab. Both patients received a 30-minute infusion.

Although higher OV doses may potentially improve viral persistence, they also increase safety concerns. In murine models, tumor elimination was recorded at the highest tolerated VV1 doses (19), and in a previous phase I study, activity signals only emerged at an intravenous dose of 1.7 × 10^11^ TCID_50_ (32), suggesting a dose threshold for clinical activity. Such a threshold may differ by tumor type, location, and susceptibility. In this study, efficacy signals were observed at the second highest dose: both patients with PRs received 1.7 × 10^10^ TCID_50_.

Our previous research demonstrated that virally encoded IFNβ is a valuable biomarker for assessing viral replication (32). Typically, IFNβ peaked 24 hours following both intratumoral and intravenous VV1, suggesting that tumoral infection may be short-lived. However, the magnitude of plasma IFNβ was highly variable between patients given the same dose of VV1. For example, in part 2, IFNβ values on day 2 ranged from 2 to 1,626 pg/mL. These differences in peak IFNβ are likely to reflect the relative differences in tumor permissiveness to infection and the patient’s immune response to VV1. Although dose strength did not seem to affect peak plasma IFNβ, infusion duration did. Peak IFNβ was significantly higher among patients infused over 30 minutes compared with patients who received 60- or 180-minute infusions. Thus a 30-minute infusion would seem to result in concentrations of VV1 that support optimal viral amplification, which, in turn, may increase the potential for improved responses.

Viral replication was tracked by NIS reporter gene imaging using ^99m^Tc pertechnetate SPECT/CT. In part 1, approximately 50% patients were NIS-positive. There was no correlation between SPECT/CT positivity and response (Supplementary Table S10).

Few clinical trials have explored the potential of OVs combined with ICIs. In a phase II trial, intratumoral T-VEC plus ipilimumab generated a significantly greater objective response rate in patients with advanced melanoma compared with ipilimumab alone (33). In a phase Ib trial, involving patients with melanoma, intratumoral T-VEC and pembrolizumab generated encouraging antitumor signals, although the subsequent phase III trial was halted because of futility (11, 34). However, recent data from the I-SPY 2 study indicate that VV1 showed a statistical improvement in PCR in the less immunoresponsive subgroups in the neoadjuvant setting. In patients with early HER2-negative breast cancer, intratumoral VV1 plus cemiplimab, followed by standard care, demonstrated an improvement over historic I-SPY data (35).

Interestingly, in both early-phase T-VEC studies that demonstrated efficacy, repeated intratumoral treatment was initiated up to 5 weeks prior to ICI initiation, whereas in the phase III trial, both treatments were administered simultaneously (11, 33, 34). In the VV1 neoadjuvant study, intratumoral treatment was administered every 3 weeks with cemiplimab up to four doses. In this study, VV1 was administered only once and on the same day as avelumab. These findings, alongside seroconversion data, indicate that the time point and frequency of OVs administration in combination with ICIs may be critical for optimal responses, and this may be a fruitful avenue for future research.

Although these results add to the developing field of OV therapy, the trial did have certain limitations. For example, tumor heterogeneity makes it difficult to predict the tumor type that may benefit from VV1. Collecting PD-(L)1, CD4, and CD8 expression data alongside NAb titers may provide clarification of the tumor types potentially susceptible to VV1 and where in the treatment pathway this novel OV may be optimally used.

In summary, this first-in-human study demonstrates that intratumoral or intravenous VV1 administration has an acceptable safety profile as monotherapy and intravenous in combination with avelumab. There is preliminary antitumor activity, now confirmed in a treatment-naïve setting**.** Intratumoral VV1 should be developed in earlier lines of therapy.

## Supplementary Material

Table S1 Injection volume, site size and image guidance criteria

Table S2 DLT definition VV1 monotherapy

Table S3 DLT definition VV1 combination therapy

Table S4 Part 1 baseline demographics

Table S5 Part 2 baseline demographics

Table S6 Part 3 baseline demographics

Table S7 Viral shedding

Table S8 VV1 neutralizing antibodies

Table S9 Part 1 disease response and SPECT/CT positivity

Table S10 Part 1 disease response and SPECT/CT positivity

Table S11 Study population representativeness

## Data Availability

The data generated in this study will be made available to qualified researchers for agreed prespecified purposes upon written request to the corresponding author.
