# Supplementary material for: Phase I Study of Oncolytic Virus VV1 as Monotherapy or in Combination with Avelumab in Patients with Relapsed Refractory Solid Tumors
Source: Cancer Res Commun. 2026 Aug 5;6(8):1862–74. doi: 10.1158/2767-9764.CRC-25-0803 (PMC13440224; doi:10.1158/2767-9764.CRC-25-0803)
Supplement: Supplementary file 1 [file crc-25-0803_table_s7_suppst7.pdf]

Listing 16.2.10.1 VSV-IFNß-NIS Plasma Concentrations
(PK/PD Population)

Cohort	Subject Number	Test (unit)	Visit	Time Point	Date/Time (Day) YYYY-MM-DDTHH:MM	Result
1: 3E6 IT	102-001	Viremia PK (copies/ug)	Day 1		2017-04-26T09:40 (1)	No (< LOD)
			Day 2		2017-04-27T09:40 (2)	No (< LOD)
			Day 3		2017-04-28T08:20 (3)	No (< LOD)
			Day 8		2017-05-03T08:20 (8)	No (< LOD)
			Day 15		2017-05-10T08:47 (15)	No (< LOD)
	102-002	Viremia PK (copies/ug)	Day 1		2017-05-03T08:30 (1)	No (< LOD)
			Day 2		2017-05-04T09:47 (2)	No (< LOD)
			Day 3		2017-05-05T11:30 (3)	No (< LOD)
			Day 8		2017-05-10T08:23 (8)	No (< LOD)
			Day 15		2017-05-17T08:09 (15)	No (< LOD)
	102-003	Viremia PK (copies/ug)	Day 1		2017-06-21T09:20 (1)	No (< LOD)
			Day 2		2017-06-22T09:21 (2)	No (< LOD)
			Day 3		2017-06-23T08:15 (3)	No (< LOD)
			Day 8		2017-06-28T08:00 (8)	No (< LOD)
			Day 15		2017-07-06T07:39 (16)	No (< LOD)
1: 1E7 IT	102-004	Viremia PK (copies/ug)	Day 1		2017-08-09T07:50 (1)	No (< LOD)
			Day 2		2017-08-10T09:00 (2)	No (< LOD)
			Day 3		2017-08-11T07:50 (3)	No (< LOD)

Cohorts are noted according to their study Part (1, 2, or 3) followed by their VSV-IFNß-NIS dosage in units of TCID50. Administration in Part 1 was IT monotherapy, in Part 2 was IV monotherapy, in Part 3 run-in and mCRC cohorts was IV combination therapy, and in Part 3 NET cohort was IV monotherapy.
Note: Subjects with shorter infusion durations than planned are grouped with the duration cohorts matching their actual infusion duration times and are noted with an asterisk.
Note: Results presented as mean +/- standard deviation.
The Limit of Detection (LOD) is 300 copies/ug.

Listing 16.2.10.1 VSV-IFNß-NIS Plasma Concentrations
(PK/PD Population)

Cohort	Subject Number	Test (unit)	Visit	Time Point	Date/Time (Day) YYYY-MM-DDTHH:MM	Result
1: 1E7 IT	102-004	Viremia PK (copies/ug)	Day 8		2017-08-16T08:01 (8)	No (< LOD)
			Day 15		2017-08-23T07:46 (15)	No (< LOD)
	104-007	Viremia PK (copies/ug)	Day 1		2017-11-01T07:02 (1)	No (< LOD)
			Day 2		2017-11-02T08:45 (2)	No (< LOD)
			Day 3		2017-11-03T13:02 (3)	No (< LOD)
			Day 8		2017-11-08T07:10 (8)	No (< LOD)
			Day 15		2017-11-13T18:00 (13)	No (< LOD)
	104-009	Viremia PK (copies/ug)	Day 1		2017-11-13T08:49 (1)	No (< LOD)
			Day 2		2017-11-14T07:54 (2)	No (< LOD)
			Day 3		2017-11-15T08:25 (3)	No (< LOD)
			Day 8		2017-11-20T15:05 (8)	No (< LOD)
			Day 15		2017-11-27T15:12 (15)	No (< LOD)
1: 3E7 IT	103-014	Viremia PK (copies/ug)	Day 1		2018-03-12T07:05 (1)	No (< LOD)
			Day 2		2018-03-13T09:32 (2)	No (< LOD)
			Day 3		2018-03-14T09:16 (3)	No (< LOD)
			Day 8		2018-03-19T10:20 (8)	No (< LOD)
			Day 15		2018-03-26T09:05 (15)	No (< LOD)
	103-015	Viremia PK (copies/ug)	Day 1		2018-03-20T08:57 (1)	No (< LOD)
			Day 2		2018-03-21T14:31 (2)	No (< LOD)

Cohorts are noted according to their study Part (1, 2, or 3) followed by their VSV-IFNß-NIS dosage in units of TCID50. Administration in Part 1 was IT monotherapy, in Part 2 was IV monotherapy, in Part 3 run-in and mCRC cohorts was IV combination therapy, and in Part 3 NET cohort was IV monotherapy.
Note: Subjects with shorter infusion durations than planned are grouped with the duration cohorts matching their actual infusion duration times and are noted with an asterisk.
Note: Results presented as mean +/- standard deviation.
The Limit of Detection (LOD) is 300 copies/ug.

Listing 16.2.10.1 VSV-IFNß-NIS Plasma Concentrations
(PK/PD Population)

Cohort	Subject Number	Test (unit)	Visit	Time Point	Date/Time (Day) YYYY-MM-DDTHH:MM	Result
1: 3E7 IT	103-015	Viremia PK (copies/ug)	Day 3		2018-03-22T12:52 (3)	No (< LOD)
			Day 8		2018-03-27T12:40 (8)	No (< LOD)
			Day 15		2018-04-03T12:28 (15)	No (< LOD)
	105-011	Viremia PK (copies/ug)	Day 1		2018-01-17T09:40 (1)	No (< LOD)
			Day 2		2018-01-18T14:00 (2)	No (< LOD)
			Day 3		2018-01-19T16:10 (3)	No (< LOD)
			Day 8		2018-01-24T10:13 (8)	No (< LOD)
			Day 15		2018-01-31T10:36 (15)	No (< LOD)
	105-012	Viremia PK (copies/ug)	Day 1		2018-02-05T15:21 (1)	No (< LOD)
			Day 2		2018-02-06T12:45 (2)	No (< LOD)
			Day 3		2018-02-07T14:32 (3)	No (< LOD)
			Day 8		2018-02-12T08:45 (8)	No (< LOD)
			Day 15		2018-02-19T08:03 (15)	No (< LOD)
1: 1E8 IT	104-017	Viremia PK (copies/ug)	Day 1		2018-04-16T07:00 (1)	No (< LOD)
			Day 2		2018-04-17T08:35 (2)	No (< LOD)
			Day 3		2018-04-18T09:10 (3)	No (< LOD)
			Day 8		2018-04-23T10:00 (8)	No (< LOD)
			Day 15		2018-04-30T11:00 (15)	No (< LOD)
	104-019	Viremia PK (copies/ug)	Day 1		2018-05-07T07:20 (1)	No (< LOD)

Cohorts are noted according to their study Part (1, 2, or 3) followed by their VSV-IFNß-NIS dosage in units of TCID50. Administration in Part 1 was IT monotherapy, in Part 2 was IV monotherapy, in Part 3 run-in and mCRC cohorts was IV combination therapy, and in Part 3 NET cohort was IV monotherapy.
Note: Subjects with shorter infusion durations than planned are grouped with the duration cohorts matching their actual infusion duration times and are noted with an asterisk.
Note: Results presented as mean +/- standard deviation.
The Limit of Detection (LOD) is 300 copies/ug.

Listing 16.2.10.1 VSV-IFNß-NIS Plasma Concentrations
(PK/PD Population)

Cohort	Subject Number	Test (unit)	Visit	Time Point	Date/Time (Day) YYYY-MM-DDTHH:MM	Result
1: 1E8 IT	104-019	Viremia PK (copies/ug)	Day 2		2018-05-08T07:15 (2)	No (< LOD)
			Day 3		2018-05-09T09:40 (3)	No (< LOD)
			Day 8		2018-05-14T09:40 (8)	No (< LOD)
			Day 15		2018-05-21T09:31 (15)	No (< LOD)
	105-016	Viremia PK (copies/ug)	Day 1		2018-04-30T11:35 (1)	No (< LOD)
			Day 2		2018-05-01T15:00 (2)	No (< LOD)
			Day 3		2018-05-02T10:42 (3)	No (< LOD)
			Day 8		2018-05-07T09:01 (8)	No (< LOD)
			Day 15		2018-05-14T10:26 (15)	No (< LOD)
	105-020	Viremia PK (copies/ug)	Day 1		2018-05-15T11:20 (1)	No (< LOD)
			Day 2		2018-05-16T15:10 (2)	No (< LOD)
			Day 3		2018-05-17T08:15 (3)	No (< LOD)
			Day 8		2018-05-22T08:18 (8)	No (< LOD)
			Day 15		2018-05-30T10:35 (16)	No (< LOD)
1: 3E8 IT	104-023	Viremia PK (copies/ug)	Day 1		2018-06-27T08:45 (1)	No (< LOD)
			Day 2		2018-06-28T08:45 (2)	No (< LOD)
			Day 3		2018-06-29T10:15 (3)	No (< LOD)
			Day 8		2018-07-03T08:58 (7)	No (< LOD)
			Day 15		2018-07-10T07:51 (14)	No (< LOD)

Cohorts are noted according to their study Part (1, 2, or 3) followed by their VSV-IFNß-NIS dosage in units of TCID50. Administration in Part 1 was IT monotherapy, in Part 2 was IV monotherapy, in Part 3 run-in and mCRC cohorts was IV combination therapy, and in Part 3 NET cohort was IV monotherapy.
Note: Subjects with shorter infusion durations than planned are grouped with the duration cohorts matching their actual infusion duration times and are noted with an asterisk.
Note: Results presented as mean +/- standard deviation.
The Limit of Detection (LOD) is 300 copies/ug.

Listing 16.2.10.1 VSV-IFNß-NIS Plasma Concentrations
(PK/PD Population)

Cohort	Subject Number	Test (unit)	Visit	Time Point	Date/Time (Day) YYYY-MM-DDTHH:MM	Result
1: 3E8 IT	104-030	Viremia PK (copies/ug)	Day 1		2018-09-12T10:40 (1)	No (< LOD)
			Day 2		2018-09-13T09:36 (2)	No (< LOD)
			Day 3		2018-09-14T12:45 (3)	No (< LOD)
			Day 8		2018-09-19T12:40 (8)	No (< LOD)
			Day 15		2018-09-26T12:35 (15)	No (< LOD)
	105-021	Viremia PK (copies/ug)	Day 1		2018-06-05T11:06 (1)	No (< LOD)
			Day 2		2018-06-06T14:10 (2)	No (< LOD)
			Day 3		2018-06-07T07:50 (3)	No (< LOD)
			Day 8		2018-06-12T14:04 (8)	No (< LOD)
			Day 15		2018-06-21T11:57 (17)	No (< LOD)
	106-022	Viremia PK (copies/ug)	Day 1		2018-06-12T09:30 (1)	No (< LOD)
			Day 2		2018-06-13T10:55 (2)	No (< LOD)
			Day 3		2018-06-14T10:48 (3)	No (< LOD)
			Day 8		2018-06-19T09:41 (8)	No (< LOD)
			Day 15		2018-06-25T09:20 (14)	No (< LOD)
1: 1E9 IT	102-028	Viremia PK (copies/ug)	Day 1		2018-09-12T07:40 (1)	No (< LOD)
			Day 2		2018-09-13T09:00 (2)	No (< LOD)
			Day 3		2018-09-14T07:44 (3)	No (< LOD)
			Day 8		2018-09-19T08:34 (8)	No (< LOD)

Cohorts are noted according to their study Part (1, 2, or 3) followed by their VSV-IFNß-NIS dosage in units of TCID50. Administration in Part 1 was IT monotherapy, in Part 2 was IV monotherapy, in Part 3 run-in and mCRC cohorts was IV combination therapy, and in Part 3 NET cohort was IV monotherapy.
Note: Subjects with shorter infusion durations than planned are grouped with the duration cohorts matching their actual infusion duration times and are noted with an asterisk.
Note: Results presented as mean +/- standard deviation.
The Limit of Detection (LOD) is 300 copies/ug.

Listing 16.2.10.1 VSV-IFNß-NIS Plasma Concentrations
(PK/PD Population)

Cohort	Subject Number	Test (unit)	Visit	Time Point	Date/Time (Day) YYYY-MM-DDTHH:MM	Result
1: 1E9 IT	102-028	Viremia PK (copies/ug)	Day 15		2018-09-26T09:47 (15)	No (< LOD)
	103-025	Viremia PK (copies/ug)	Day 1		2018-07-31T07:30 (1)	No (< LOD)
			Day 2		2018-08-01T16:24 (2)	No (< LOD)
			Day 3		2018-08-02T13:50 (3)	No (< LOD)
			Day 8		2018-08-07T09:30 (8)	No (< LOD)
			Day 15		2018-08-14T10:15 (15)	No (< LOD)
	103-029	Viremia PK (copies/ug)	Day 1		2018-09-05T07:20 (1)	No (< LOD)
			Day 2		2018-09-06T12:48 (2)	No (< LOD)
			Day 3		2018-09-07T14:45 (3)	No (< LOD)
			Day 8		2018-09-12T12:44 (8)	No (< LOD)
			Day 15		2018-09-19T12:06 (15)	No (< LOD)
	105-026	Viremia PK (copies/ug)	Day 1		2018-08-13T09:50 (1)	No (< LOD)
			Day 2		2018-08-14T14:00 (2)	No (< LOD)
			Day 3		2018-08-15T08:35 (3)	No (< LOD)
			Day 8		2018-08-20T09:55 (8)	No (< LOD)
			Day 15		2018-08-29T08:15 (17)	No (< LOD)
	105-027	Viremia PK (copies/ug)	Day 1		2018-08-20T11:58 (1)	No (< LOD)
			Day 2		2018-08-21T16:00 (2)	No (< LOD)
			Day 3		2018-08-22T08:29 (3)	No (< LOD)
			Day 8		2018-08-27T10:28 (8)	No (< LOD)

Cohorts are noted according to their study Part (1, 2, or 3) followed by their VSV-IFNß-NIS dosage in units of TCID50. Administration in Part 1 was IT monotherapy, in Part 2 was IV monotherapy, in Part 3 run-in and mCRC cohorts was IV combination therapy, and in Part 3 NET cohort was IV monotherapy.
Note: Subjects with shorter infusion durations than planned are grouped with the duration cohorts matching their actual infusion duration times and are noted with an asterisk.
Note: Results presented as mean +/- standard deviation.
The Limit of Detection (LOD) is 300 copies/ug.

Listing 16.2.10.1 VSV-IFNß-NIS Plasma Concentrations
(PK/PD Population)

Cohort	Subject Number	Test (unit)	Visit	Time Point	Date/Time (Day) YYYY-MM-DDTHH:MM	Result
1: 1E9 IT	105-027	Viremia PK (copies/ug)	Day 15		2018-09-05T09:25 (17)	No (< LOD)
	106-032	Viremia PK (copies/ug)	Day 1		2018-10-16T08:34 (1)	No (< LOD)
			Day 2		2018-10-17T08:50 (2)	No (< LOD)
			Day 3		2018-10-18T11:40 (3)	No (< LOD)
			Day 8		2018-10-23T10:10 (8)	No (< LOD)
			Day 15		2018-10-29T11:00 (14)	No (< LOD)
1: 3E9 IT	102-033	Viremia PK (copies/ug)	Day 1		2018-11-19T08:19 (1)	No (< LOD)
			Day 2		2018-11-20T07:43 (2)	49600 +/- 3270
			Day 3		2018-11-21T09:23 (3)	No (< LOD)
			Day 8		2018-11-26T09:45 (8)	No (< LOD)
			Day 15		2018-12-03T09:26 (15)	No (< LOD)
	104-039	Viremia PK (copies/ug)	Day 1		2019-01-02T08:45 (1)	No (< LOD)
			Day 2		2019-01-03T09:11 (2)	No (< LOD)
			Day 3		2019-01-04T09:00 (3)	No (< LOD)
			Day 8		2019-01-09T17:40 (8)	No (< LOD)
			Day 15		2019-01-17T15:28 (16)	No (< LOD)
	105-038	Viremia PK (copies/ug)	Day 1		2018-12-03T11:13 (1)	No (< LOD)
			Day 2		2018-12-04T16:15 (2)	No (< LOD)
			Day 3		2018-12-05T08:47 (3)	No (< LOD)
			Day 8		2018-12-10T08:40 (8)	No (< LOD)

Cohorts are noted according to their study Part (1, 2, or 3) followed by their VSV-IFNß-NIS dosage in units of TCID50. Administration in Part 1 was IT monotherapy, in Part 2 was IV monotherapy, in Part 3 run-in and mCRC cohorts was IV combination therapy, and in Part 3 NET cohort was IV monotherapy.
Note: Subjects with shorter infusion durations than planned are grouped with the duration cohorts matching their actual infusion duration times and are noted with an asterisk.
Note: Results presented as mean +/- standard deviation.
The Limit of Detection (LOD) is 300 copies/ug.

Listing 16.2.10.1 VSV-IFNß-NIS Plasma Concentrations
(PK/PD Population)

Cohort	Subject Number	Test (unit)	Visit	Time Point	Date/Time (Day) YYYY-MM-DDTHH:MM	Result
1: 3E9 IT	105-038	Viremia PK (copies/ug)	Day 15		2018-12-19T08:47 (17)	No (< LOD)
2: 1.7E10 15m	102-061*	Viremia PK (copies/ug)	Day 1	Pre-Infusion	2020-01-21T08:33 (1)	No (< LOD)
			Day 1	End of Infusion	2020-01-21T11:53 (1)	94200000 +/- 456000
			Day 1	4 Hrs Post Infusion	2020-01-21T15:53 (1)	282000 +/- 4250
			Day 2		2020-01-22T11:45 (2)	323000 +/- 22900
			Day 8		2020-01-28T08:45 (8)	No (< LOD)
			Day 15		2020-02-06T09:02 (17)	No (< LOD)
	102-062	Viremia PK (copies/ug)	Day 1	Pre-Infusion	2020-01-22T07:45 (1)	No (< LOD)
			Day 1	End of Infusion	2020-01-22T10:50 (1)	926000 +/- 196000
			Day 1	4 Hrs Post Infusion	2020-01-22T14:42 (1)	2400 +/- 925
			Day 2		2020-01-23T10:46 (2)	1990 +/- 455
			Day 8		2020-01-29T07:31 (8)	No (< LOD)
			Day 15		2020-02-04T07:42 (14)	No (< LOD)
	103-070	Viremia PK (copies/ug)	Day 1	Pre-Infusion	2020-06-09T10:46 (1)	No (< LOD)
			Day 1	End of Infusion	2020-06-09T12:23 (1)	246000 +/- 36400
			Day 1	4 Hrs Post Infusion	2020-06-09T16:10 (1)	No (< LOD)
			Day 2		2020-06-10T10:42 (2)	No (< LOD)
			Day 8		2020-06-16T08:31 (8)	No (< LOD)
	104-067	Viremia PK (copies/ug)	Day 1	Pre-Infusion	2020-03-24T10:40 (1)	No (< LOD)

Cohorts are noted according to their study Part (1, 2, or 3) followed by their VSV-IFNß-NIS dosage in units of TCID50. Administration in Part 1 was IT monotherapy, in Part 2 was IV monotherapy, in Part 3 run-in and mCRC cohorts was IV combination therapy, and in Part 3 NET cohort was IV monotherapy.
Note: Subjects with shorter infusion durations than planned are grouped with the duration cohorts matching their actual infusion duration times and are noted with an asterisk.
Note: Results presented as mean +/- standard deviation.
The Limit of Detection (LOD) is 300 copies/ug.

Listing 16.2.10.1 VSV-IFNß-NIS Plasma Concentrations
(PK/PD Population)

Cohort	Subject Number	Test (unit)	Visit	Time Point	Date/Time (Day) YYYY-MM-DDTHH:MM	Result
2: 1.7E10 15m	104-067	Viremia PK (copies/ug)	Day 1	End of Infusion	2020-03-24T15:45 (1)	28400 +/- 2230
			Day 1	4 Hrs Post Infusion	2020-03-24T18:45 (1)	519 +/- 60.3
			Day 2		2020-03-25T13:40 (2)	No (< LOD)
			Day 8		2020-03-31T12:52 (8)	No (< LOD)
			Day 15		2020-04-07T13:11 (15)	No (< LOD)
	105-063	Viremia PK (copies/ug)	Day 1	Pre-Infusion	2020-01-29T09:28 (1)	No (< LOD)
			Day 1	End of Infusion	2020-01-29T17:38 (1)	766000 +/- 84500
			Day 1	4 Hrs Post Infusion	2020-01-29T21:37 (1)	No (< LOD)
			Day 2		2020-01-30T16:17 (2)	No (< LOD)
			Day 8		2020-02-05T08:44 (8)	No (< LOD)
			Day 15		2020-02-12T09:27 (15)	No (< LOD)
2: 1.7E10 30m	102-044	Viremia PK (copies/ug)	Day 1		2019-04-08T10:00 (1)	No (< LOD)
			Day 2		2019-04-09T09:54 (2)	No (< LOD)
			Day 3		2019-04-10T08:39 (3)	No (< LOD)
			Day 8		2019-04-15T09:37 (8)	No (< LOD)
			Day 15		2019-04-22T09:59 (15)	No (< LOD)
	103-046	Viremia PK (copies/ug)	Day 1		2019-04-15T07:35 (1)	No (< LOD)
			Day 2		2019-04-16T10:00 (2)	No (< LOD)
			Day 3		2019-04-17T15:45 (3)	No (< LOD)
			Day 8		2019-04-22T11:44 (8)	No (< LOD)

Cohorts are noted according to their study Part (1, 2, or 3) followed by their VSV-IFNß-NIS dosage in units of TCID50. Administration in Part 1 was IT monotherapy, in Part 2 was IV monotherapy, in Part 3 run-in and mCRC cohorts was IV combination therapy, and in Part 3 NET cohort was IV monotherapy.
Note: Subjects with shorter infusion durations than planned are grouped with the duration cohorts matching their actual infusion duration times and are noted with an asterisk.
Note: Results presented as mean +/- standard deviation.
The Limit of Detection (LOD) is 300 copies/ug.

Listing 16.2.10.1 VSV-IFNß-NIS Plasma Concentrations
(PK/PD Population)

Cohort	Subject Number	Test (unit)	Visit	Time Point	Date/Time (Day) YYYY-MM-DDTHH:MM	Result
2: 1.7E10 30m	103-046	Viremia PK (copies/ug)	Day 15		2019-04-29T10:47 (15)	No (< LOD)
	103-060*	Viremia PK (copies/ug)	Day 1		2019-07-31T08:39 (1)	No (< LOD)
			Day 2		2019-08-01T08:31 (2)	No (< LOD)
			Day 3		2019-08-02T08:45 (3)	No (< LOD)
			Day 8		2019-08-07T10:50 (8)	No (< LOD)
			Day 15		2019-08-14T11:18 (15)	No (< LOD)
	105-040	Viremia PK (copies/ug)	Day 1		2019-02-11T11:00 (1)	No (< LOD)
			Day 2		2019-02-12T12:30 (2)	2060 +/- 189
			Day 3		2019-02-13T10:30 (3)	No (< LOD)
			Day 8		2019-02-18T10:00 (8)	No (< LOD)
			Day 15		2019-02-27T14:37 (17)	No (< LOD)
	105-041	Viremia PK (copies/ug)	Day 1		2019-02-18T12:55 (1)	No (< LOD)
			Day 2		2019-02-19T12:30 (2)	4600000 +/- 82400
			Day 3		2019-02-20T10:41 (3)	38000 +/- 2050
			Day 8		2019-02-25T09:19 (8)	No (< LOD)
			Day 15		2019-03-06T10:00 (17)	No (< LOD)
	105-042	Viremia PK (copies/ug)	Day 1		2019-03-18T10:03 (1)	No (< LOD)
			Day 2		2019-03-19T11:45 (2)	9930 +/- 360
			Day 3		2019-03-20T10:18 (3)	19500 +/- 2170
			Day 8		2019-03-25T09:13 (8)	13800 +/- 785

Cohorts are noted according to their study Part (1, 2, or 3) followed by their VSV-IFNß-NIS dosage in units of TCID50. Administration in Part 1 was IT monotherapy, in Part 2 was IV monotherapy, in Part 3 run-in and mCRC cohorts was IV combination therapy, and in Part 3 NET cohort was IV monotherapy.
Note: Subjects with shorter infusion durations than planned are grouped with the duration cohorts matching their actual infusion duration times and are noted with an asterisk.
Note: Results presented as mean +/- standard deviation.
The Limit of Detection (LOD) is 300 copies/ug.

Listing 16.2.10.1 VSV-IFNß-NIS Plasma Concentrations
(PK/PD Population)

Cohort	Subject Number	Test (unit)	Visit	Time Point	Date/Time (Day) YYYY-MM-DDTHH:MM	Result
2: 1.7E10 30m	105-042	Viremia PK (copies/ug)	Day 15		2019-04-03T09:25 (17)	No (< LOD)
	106-043	Viremia PK (copies/ug)	Day 1		2019-04-08T09:45 (1)	No (< LOD)
			Day 2		2019-04-09T09:08 (2)	No (< LOD)
			Day 3		2019-04-10T09:43 (3)	No (< LOD)
			Day 8		2019-04-15T08:55 (8)	No (< LOD)
			Day 15		2019-04-22T10:40 (15)	No (< LOD)
2: 1.7E10 60m	102-057	Viremia PK (copies/ug)	Day 1		2019-07-01T09:20 (1)	No (< LOD)
			Day 2		2019-07-02T09:40 (2)	No (< LOD)
			Day 3		2019-07-03T10:11 (3)	No (< LOD)
			Day 8		2019-07-08T08:19 (8)	No (< LOD)
			Day 15		2019-07-15T10:48 (15)	No (< LOD)
	102-058	Viremia PK (copies/ug)	Day 1		2019-07-01T08:49 (1)	No (< LOD)
			Day 2		2019-07-02T10:02 (2)	No (< LOD)
			Day 3		2019-07-03T09:43 (3)	No (< LOD)
			Day 8		2019-07-08T08:05 (8)	No (< LOD)
			Day 15		2019-07-15T10:35 (15)	No (< LOD)
	104-056	Viremia PK (copies/ug)	Day 1		2019-06-19T08:55 (1)	No (< LOD)
			Day 2		2019-06-20T10:20 (2)	41200 +/- 6380
			Day 3		2019-06-21T11:20 (3)	No (< LOD)

Cohorts are noted according to their study Part (1, 2, or 3) followed by their VSV-IFNß-NIS dosage in units of TCID50. Administration in Part 1 was IT monotherapy, in Part 2 was IV monotherapy, in Part 3 run-in and mCRC cohorts was IV combination therapy, and in Part 3 NET cohort was IV monotherapy.
Note: Subjects with shorter infusion durations than planned are grouped with the duration cohorts matching their actual infusion duration times and are noted with an asterisk.
Note: Results presented as mean +/- standard deviation.
The Limit of Detection (LOD) is 300 copies/ug.

Listing 16.2.10.1 VSV-IFNß-NIS Plasma Concentrations
(PK/PD Population)

Cohort	Subject Number	Test (unit)	Visit	Time Point	Date/Time (Day) YYYY-MM-DDTHH:MM	Result
2: 1.7E10 60m	104-056	Viremia PK (copies/ug)	Day 8		2019-06-26T09:20 (8)	No (< LOD)
			Day 15		2019-07-03T14:05 (15)	No (< LOD)
	105-055	Viremia PK (copies/ug)	Day 1		2019-06-10T07:56 (1)	No (< LOD)
			Day 2		2019-06-11T13:15 (2)	117000 +/- 36700
			Day 3		2019-06-12T09:06 (3)	No (< LOD)
			Day 8		2019-06-17T08:24 (8)	No (< LOD)
			Day 15		2019-06-26T08:11 (17)	No (< LOD)
	105-059	Viremia PK (copies/ug)	Day 1		2019-07-15T15:30 (1)	No (< LOD)
			Day 2		2019-07-16T15:30 (2)	No (< LOD)
			Day 3		2019-07-17T14:40 (3)	No (< LOD)
			Day 8		2019-07-22T11:30 (8)	No (< LOD)
			Day 15		2019-07-31T10:46 (17)	No (< LOD)
2: 1.7E10 180m	102-048	Viremia PK (copies/ug)	Day 1		2019-04-29T09:45 (1)	No (< LOD)
			Day 2		2019-04-30T13:14 (2)	167000 +/- 12600
			Day 3		2019-05-01T09:52 (3)	44500 +/- 3270
			Day 8		2019-05-06T13:09 (8)	No (< LOD)
			Day 15		2019-05-14T14:15 (16)	No (< LOD)
	102-053	Viremia PK (copies/ug)	Day 1		2019-05-14T10:22 (1)	No (< LOD)
			Day 2		2019-05-15T09:10 (2)	No (< LOD)

Cohorts are noted according to their study Part (1, 2, or 3) followed by their VSV-IFNß-NIS dosage in units of TCID50. Administration in Part 1 was IT monotherapy, in Part 2 was IV monotherapy, in Part 3 run-in and mCRC cohorts was IV combination therapy, and in Part 3 NET cohort was IV monotherapy.
Note: Subjects with shorter infusion durations than planned are grouped with the duration cohorts matching their actual infusion duration times and are noted with an asterisk.
Note: Results presented as mean +/- standard deviation.
The Limit of Detection (LOD) is 300 copies/ug.

Listing 16.2.10.1 VSV-IFNß-NIS Plasma Concentrations
(PK/PD Population)

Cohort	Subject Number	Test (unit)	Visit	Time Point	Date/Time (Day) YYYY-MM-DDTHH:MM	Result
2: 1.7E10 180m	102-053	Viremia PK (copies/ug)	Day 3		2019-05-16T07:54 (3)	No (< LOD)
			Day 8		2019-05-21T10:16 (8)	No (< LOD)
			Day 15		2019-05-30T10:20 (17)	No (< LOD)
	102-054	Viremia PK (copies/ug)	Day 1		2019-05-29T09:17 (1)	No (< LOD)
			Day 2		2019-05-30T08:45 (2)	No (< LOD)
			Day 3		2019-05-31T12:23 (3)	No (< LOD)
			Day 8		2019-06-05T09:45 (8)	No (< LOD)
			Day 15		2019-06-13T09:35 (16)	No (< LOD)
	104-047	Viremia PK (copies/ug)	Day 1		2019-04-23T08:20 (1)	No (< LOD)
			Day 2		2019-04-24T11:00 (2)	3740 +/- 277
			Day 3		2019-04-25T09:15 (3)	No (< LOD)
			Day 8		2019-04-30T10:55 (8)	No (< LOD)
			Day 15		2019-05-07T09:47 (15)	No (< LOD)
	104-050	Viremia PK (copies/ug)	Day 1		2019-05-01T10:45 (1)	No (< LOD)
			Day 2		2019-05-02T10:00 (2)	6820 +/- 1040
			Day 3		2019-05-03T08:40 (3)	84800 +/- 6450
			Day 8		2019-05-08T16:00 (8)	1100 +/- 140
			Day 15		2019-05-15T14:13 (15)	1200 +/- 0.422
			Day 22		2019-05-22T11:15 (22)	No (< LOD)
	105-052	Viremia PK (copies/ug)	Day 1		2019-05-28T08:40 (1)	No (< LOD)

Cohorts are noted according to their study Part (1, 2, or 3) followed by their VSV-IFNß-NIS dosage in units of TCID50. Administration in Part 1 was IT monotherapy, in Part 2 was IV monotherapy, in Part 3 run-in and mCRC cohorts was IV combination therapy, and in Part 3 NET cohort was IV monotherapy.
Note: Subjects with shorter infusion durations than planned are grouped with the duration cohorts matching their actual infusion duration times and are noted with an asterisk.
Note: Results presented as mean +/- standard deviation.
The Limit of Detection (LOD) is 300 copies/ug.

Listing 16.2.10.1 VSV-IFNß-NIS Plasma Concentrations
(PK/PD Population)

Cohort	Subject Number	Test (unit)	Visit	Time Point	Date/Time (Day) YYYY-MM-DDTHH:MM	Result
2: 1.7E10 180m	105-052	Viremia PK (copies/ug)	Day 2		2019-05-29T15:25 (2)	No (< LOD)
			Day 3		2019-05-30T10:47 (3)	No (< LOD)
			Day 8		2019-06-04T10:04 (8)	No (< LOD)
			Day 15		2019-06-12T10:21 (16)	No (< LOD)
2: 1.0E11 MTD	102-093	Viremia PK (copies/ug)	Day 1	Pre-Infusion	2021-04-26T12:22 (1)	No (< LOD)
			Day 1	End of Infusion	2021-04-26T13:22 (1)	214000 +/- 26000
			Day 1	4 Hrs Post Infusion	2021-04-26T17:15 (1)	81800000 +/- 11400000
			Day 2		2021-04-27T11:10 (2)	45300 +/- 12100
			Day 8		2021-05-03T12:00 (8)	1980 +/- 490
			Day 15		2021-05-10T09:16 (15)	No (< LOD)
	104-095	Viremia PK (copies/ug)	Day 1	Pre-Infusion	2021-04-26T09:05 (1)	No (< LOD)
			Day 1	End of Infusion	2021-04-26T12:05 (1)	28000000 +/- 5880000
			Day 1	4 Hrs Post Infusion	2021-04-26T16:00 (1)	17400 +/- 2260
			Day 2		2021-04-27T11:33 (2)	14800 +/- 90.8
			Day 8		2021-05-03T14:02 (8)	No (< LOD)
			Day 15		2021-05-12T10:50 (17)	No (< LOD)
	104-096	Viremia PK (copies/ug)	Day 1	Pre-Infusion	2021-05-04T09:55 (1)	No (< LOD)
			Day 1	End of Infusion	2021-05-04T13:04 (1)	No (< LOD)
			Day 1	4 Hrs Post Infusion	2021-05-04T16:56 (1)	No (< LOD)
			Day 2		2021-05-05T13:10 (2)	No (< LOD)

Cohorts are noted according to their study Part (1, 2, or 3) followed by their VSV-IFNß-NIS dosage in units of TCID50. Administration in Part 1 was IT monotherapy, in Part 2 was IV monotherapy, in Part 3 run-in and mCRC cohorts was IV combination therapy, and in Part 3 NET cohort was IV monotherapy.
Note: Subjects with shorter infusion durations than planned are grouped with the duration cohorts matching their actual infusion duration times and are noted with an asterisk.
Note: Results presented as mean +/- standard deviation.
The Limit of Detection (LOD) is 300 copies/ug.

Listing 16.2.10.1 VSV-IFNß-NIS Plasma Concentrations
(PK/PD Population)

Cohort	Subject Number	Test (unit)	Visit	Time Point	Date/Time (Day) YYYY-MM-DDTHH:MM	Result
2: 1.0E11 MTD	104-096	Viremia PK (copies/ug)	Day 8		2021-05-11T13:27 (8)	No (< LOD)
			Day 15		2021-05-19T09:05 (16)	No (< LOD)
	104-097	Viremia PK (copies/ug)	Day 1	Pre-Infusion	2021-05-18T10:30 (1)	No (< LOD)
			Day 1	End of Infusion	2021-05-18T13:30 (1)	16100000 +/- 3060000
			Day 1	4 Hrs Post Infusion	2021-05-18T17:00 (1)	8830000 +/- 2010000
			Day 2		2021-05-19T13:00 (2)	172000 +/- 23500
			Day 8		2021-05-25T12:30 (8)	No (< LOD)
			Day 15		2021-06-02T10:51 (16)	No (< LOD)
	104-098	Viremia PK (copies/ug)	Day 1	Pre-Infusion	2021-05-10T09:20 (1)	No (< LOD)
			Day 1	End of Infusion	2021-05-10T12:10 (1)	No (< LOD)
			Day 1	4 Hrs Post Infusion	2021-05-10T16:11 (1)	No (< LOD)
			Day 2		2021-05-11T11:20 (2)	No (< LOD)
			Day 8		2021-05-17T14:00 (8)	No (< LOD)
			Day 15		2021-05-26T11:15 (17)	No (< LOD)
	104-099	Viremia PK (copies/ug)	Day 1	Pre-Infusion	2021-06-01T11:16 (1)	No (< LOD)
			Day 1	End of Infusion	2021-06-01T12:00 (1)	3780000 +/- 330000
			Day 1	4 Hrs Post Infusion	2021-06-01T16:06 (1)	338000 +/- 66300
			Day 2		2021-06-02T11:58 (2)	3330 +/- 0
			Day 8		2021-06-08T13:20 (8)	No (< LOD)
			Day 15		2021-06-16T09:19 (16)	No (< LOD)

Cohorts are noted according to their study Part (1, 2, or 3) followed by their VSV-IFNß-NIS dosage in units of TCID50. Administration in Part 1 was IT monotherapy, in Part 2 was IV monotherapy, in Part 3 run-in and mCRC cohorts was IV combination therapy, and in Part 3 NET cohort was IV monotherapy.
Note: Subjects with shorter infusion durations than planned are grouped with the duration cohorts matching their actual infusion duration times and are noted with an asterisk.
Note: Results presented as mean +/- standard deviation.
The Limit of Detection (LOD) is 300 copies/ug.

Listing 16.2.10.1 VSV-IFNß-NIS Plasma Concentrations
(PK/PD Population)

Cohort	Subject Number	Test (unit)	Visit	Time Point	Date/Time (Day) YYYY-MM-DDTHH:MM	Result
3: 1.7E10 Run-In	104-068	Viremia PK (copies/ug)	C1-Day 1	Pre-Infusion	2020-03-23T09:52 (1)	No (< LOD)
			C1-Day 1	End of Infusion	2020-03-23T16:17 (1)	3940 +/- 1450
			C1-Day 1	4 Hrs Post Infusion	2020-03-23T20:00 (1)	240 +/- 69.9
			C1-Day 2		2020-03-24T13:37 (2)	535 +/- 85.6
			C1-Day 8		2020-03-30T10:02 (8)	No (< LOD)
			C1-Day 15		2020-04-06T12:22 (15)	No (< LOD)
	105-065	Viremia PK (copies/ug)	C1-Day 1	Pre-Infusion	2020-02-18T15:02 (1)	No (< LOD)
			C1-Day 1	End of Infusion	2020-02-18T16:18 (1)	2010000 +/- 51600
			C1-Day 1	4 Hrs Post Infusion	2020-02-18T20:35 (1)	69400 +/- 5620
			C1-Day 2		2020-02-19T15:26 (2)	30900 +/- 1160
			C1-Day 8		2020-02-25T09:57 (8)	No (< LOD)
			C1-Day 15		2020-03-05T13:15 (17)	No (< LOD)
	105-066	Viremia PK (copies/ug)	C1-Day 1	Pre-Infusion	2020-02-26T11:05 (1)	No (< LOD)
			C1-Day 1	End of Infusion	2020-02-26T13:20 (1)	332000 +/- 11400
			C1-Day 1	4 Hrs Post Infusion	2020-02-26T17:20 (1)	No (< LOD)
			C1-Day 2		2020-02-27T12:50 (2)	No (< LOD)
			C1-Day 8		2020-03-04T12:16 (8)	No (< LOD)
			C1-Day 15		2020-03-11T12:15 (15)	No (< LOD)
3: 1.7E10 mCRC	102-071	Viremia PK (copies/ug)	C1-Day 1	Pre-Infusion	2020-07-28T07:39 (1)	No (< LOD)

Cohorts are noted according to their study Part (1, 2, or 3) followed by their VSV-IFNß-NIS dosage in units of TCID50. Administration in Part 1 was IT monotherapy, in Part 2 was IV monotherapy, in Part 3 run-in and mCRC cohorts was IV combination therapy, and in Part 3 NET cohort was IV monotherapy.
Note: Subjects with shorter infusion durations than planned are grouped with the duration cohorts matching their actual infusion duration times and are noted with an asterisk.
Note: Results presented as mean +/- standard deviation.
The Limit of Detection (LOD) is 300 copies/ug.

Listing 16.2.10.1 VSV-IFNß-NIS Plasma Concentrations
(PK/PD Population)

Cohort	Subject Number	Test (unit)	Visit	Time Point	Date/Time (Day) YYYY-MM-DDTHH:MM	Result
3: 1.7E10 mCRC	102-071	Viremia PK (copies/ug)	C1-Day 1	End of Infusion	2020-07-28T12:10 (1)	4860 +/- 1870
			C1-Day 1	4 Hrs Post Infusion	2020-07-28T16:00 (1)	No (< LOD)
			C1-Day 2		2020-07-29T12:02 (2)	No (< LOD)
			C1-Day 8		2020-08-04T08:18 (8)	No (< LOD)
			C1-Day 15		2020-08-10T10:03 (14)	No (< LOD)
	102-080	Viremia PK (copies/ug)	C1-Day 1	Pre-Infusion	2020-10-20T10:24 (1)	No (< LOD)
			C1-Day 1	End of Infusion	2020-10-20T12:17 (1)	290000 +/- 21400
			C1-Day 1	4 Hrs Post Infusion	2020-10-20T16:12 (1)	1240 +/- 99.3
			C1-Day 2		2020-10-21T10:55 (2)	No (< LOD)
			C1-Day 8		2020-10-27T12:18 (8)	No (< LOD)
			C1-Day 15		2020-11-02T10:22 (14)	No (< LOD)
	103-076	Viremia PK (copies/ug)	C1-Day 1	Pre-Infusion	2020-09-21T08:20 (1)	No (< LOD)
			C1-Day 1	End of Infusion	2020-09-21T09:58 (1)	66500 +/- 14700
			C1-Day 1	4 Hrs Post Infusion	2020-09-21T13:47 (1)	No (< LOD)
			C1-Day 2		2020-09-22T08:35 (2)	No (< LOD)
			C1-Day 8		2020-09-30T12:57 (10)	No (< LOD)
			C1-Day 15		2020-10-05T12:08 (15)	No (< LOD)
	103-081	Viremia PK (copies/ug)	C1-Day 1	Pre-Infusion	2020-10-12T07:40 (1)	No (< LOD)
			C1-Day 1	End of Infusion	2020-10-12T09:50 (1)	33200 +/- 3820
			C1-Day 1	4 Hrs Post Infusion	2020-10-12T13:46 (1)	No (< LOD)
			C1-Day 2		2020-10-13T09:30 (2)	No (< LOD)

Cohorts are noted according to their study Part (1, 2, or 3) followed by their VSV-IFNß-NIS dosage in units of TCID50. Administration in Part 1 was IT monotherapy, in Part 2 was IV monotherapy, in Part 3 run-in and mCRC cohorts was IV combination therapy, and in Part 3 NET cohort was IV monotherapy.
Note: Subjects with shorter infusion durations than planned are grouped with the duration cohorts matching their actual infusion duration times and are noted with an asterisk.
Note: Results presented as mean +/- standard deviation.
The Limit of Detection (LOD) is 300 copies/ug.

Listing 16.2.10.1 VSV-IFNß-NIS Plasma Concentrations
(PK/PD Population)

Cohort	Subject Number	Test (unit)	Visit	Time Point	Date/Time (Day) YYYY-MM-DDTHH:MM	Result
3: 1.7E10 mCRC	103-081	Viremia PK (copies/ug)	C1-Day 8		2020-10-19T14:10 (8)	No (< LOD)
			C1-Day 15		2020-10-26T11:32 (15)	No (< LOD)
	103-082	Viremia PK (copies/ug)	C1-Day 1	Pre-Infusion	2020-10-19T09:02 (1)	No (< LOD)
			C1-Day 1	End of Infusion	2020-10-19T11:23 (1)	99000 +/- 14900
			C1-Day 1	4 Hrs Post Infusion	2020-10-19T15:09 (1)	No (< LOD)
			C1-Day 2		2020-10-20T11:24 (2)	No (< LOD)
			C1-Day 8		2020-10-26T10:51 (8)	No (< LOD)
			C1-Day 15		2020-11-02T10:36 (15)	No (< LOD)
	103-084	Viremia PK (copies/ug)	C1-Day 1	Pre-Infusion	2020-11-16T10:23 (1)	No (< LOD)
			C1-Day 1	End of Infusion	2020-11-16T11:00 (1)	5070000 +/- 352000
			C1-Day 1	4 Hrs Post Infusion	2020-11-16T14:58 (1)	No (< LOD)
			C1-Day 2		2020-11-17T11:15 (2)	No (< LOD)
			C1-Day 8		2020-11-23T14:15 (8)	No (< LOD)
			C1-Day 15		2020-11-30T11:40 (15)	No (< LOD)
	103-086	Viremia PK (copies/ug)	C1-Day 1	Pre-Infusion	2020-11-09T09:42 (1)	No (< LOD)
			C1-Day 1	End of Infusion	2020-11-09T11:08 (1)	34600 +/- 4410
			C1-Day 1	4 Hrs Post Infusion	2020-11-09T14:57 (1)	No (< LOD)
			C1-Day 2		2020-11-10T12:01 (2)	No (< LOD)
			C1-Day 8		2020-11-16T11:35 (8)	No (< LOD)
			C1-Day 15		2020-11-23T12:09 (15)	No (< LOD)

Cohorts are noted according to their study Part (1, 2, or 3) followed by their VSV-IFNß-NIS dosage in units of TCID50. Administration in Part 1 was IT monotherapy, in Part 2 was IV monotherapy, in Part 3 run-in and mCRC cohorts was IV combination therapy, and in Part 3 NET cohort was IV monotherapy.
Note: Subjects with shorter infusion durations than planned are grouped with the duration cohorts matching their actual infusion duration times and are noted with an asterisk.
Note: Results presented as mean +/- standard deviation.
The Limit of Detection (LOD) is 300 copies/ug.

Listing 16.2.10.1 VSV-IFNß-NIS Plasma Concentrations
(PK/PD Population)

Cohort	Subject Number	Test (unit)	Visit	Time Point	Date/Time (Day) YYYY-MM-DDTHH:MM	Result
3: 1.7E10 mCRC	103-088	Viremia PK (copies/ug)	C1-Day 1	Pre-Infusion	2020-11-17T07:28 (1)	No (< LOD)
			C1-Day 1	End of Infusion	2020-11-17T09:50 (1)	768000 +/- 77300
			C1-Day 1	4 Hrs Post Infusion	2020-11-17T13:50 (1)	7070 +/- 616
			C1-Day 2		2020-11-18T11:05 (2)	No (< LOD)
			C1-Day 8		2020-11-24T12:50 (8)	No (< LOD)
			C1-Day 15		2020-12-01T13:10 (15)	No (< LOD)
	104-089	Viremia PK (copies/ug)	C1-Day 1	Pre-Infusion	2021-01-12T10:45 (1)	No (< LOD)
			C1-Day 1	End of Infusion	2021-01-12T13:45 (1)	No (< LOD)
			C1-Day 1	4 Hrs Post Infusion	2021-01-12T16:25 (1)	No (< LOD)
			C1-Day 2		2021-01-13T11:50 (2)	No (< LOD)
			C1-Day 8		2021-01-19T07:50 (8)	No (< LOD)
			C1-Day 15		2021-01-26T07:30 (15)	No (< LOD)
	104-090	Viremia PK (copies/ug)	C1-Day 1	Pre-Infusion	2021-01-19T13:25 (1)	No (< LOD)
			C1-Day 1	End of Infusion	2021-01-19T16:10 (1)	709000 +/- 3220
			C1-Day 1	4 Hrs Post Infusion	2021-01-19T20:13 (1)	No (< LOD)
			C1-Day 8		2021-01-26T13:05 (8)	No (< LOD)
			C1-Day 15		2021-02-02T08:11 (15)	No (< LOD)
	105-085	Viremia PK (copies/ug)	C1-Day 1	Pre-Infusion	2020-11-04T10:54 (1)	No (< LOD)
			C1-Day 1	End of Infusion	2020-11-04T13:03 (1)	151000 +/- 15400
			C1-Day 1	4 Hrs Post Infusion	2020-11-04T17:04 (1)	No (< LOD)
			C1-Day 2		2020-11-05T12:55 (2)	No (< LOD)

Cohorts are noted according to their study Part (1, 2, or 3) followed by their VSV-IFNß-NIS dosage in units of TCID50. Administration in Part 1 was IT monotherapy, in Part 2 was IV monotherapy, in Part 3 run-in and mCRC cohorts was IV combination therapy, and in Part 3 NET cohort was IV monotherapy.
Note: Subjects with shorter infusion durations than planned are grouped with the duration cohorts matching their actual infusion duration times and are noted with an asterisk.
Note: Results presented as mean +/- standard deviation.
The Limit of Detection (LOD) is 300 copies/ug.

Listing 16.2.10.1 VSV-IFNß-NIS Plasma Concentrations
(PK/PD Population)

Cohort	Subject Number	Test (unit)	Visit	Time Point	Date/Time (Day) YYYY-MM-DDTHH:MM	Result
3: 1.7E10 mCRC	105-085	Viremia PK (copies/ug)	C1-Day 8		2020-11-11T08:10 (8)	No (< LOD)
			C1-Day 15		2020-11-18T12:30 (15)	No (< LOD)
	110-072	Viremia PK (copies/ug)	C1-Day 1	Pre-Infusion	2020-08-26T09:20 (1)	No (< LOD)
			C1-Day 1	End of Infusion	2020-08-26T10:20 (1)	20500 +/- 802
			C1-Day 1	4 Hrs Post Infusion	2020-08-26T14:18 (1)	No (< LOD)
			C1-Day 2		2020-08-27T10:18 (2)	No (< LOD)
			C1-Day 8		2020-09-02T12:55 (8)	No (< LOD)
	110-075	Viremia PK (copies/ug)	C1-Day 1	Pre-Infusion	2020-08-25T10:00 (1)	No (< LOD)
			C1-Day 1	End of Infusion	2020-08-25T10:58 (1)	15000 +/- 953
			C1-Day 1	4 Hrs Post Infusion	2020-08-25T14:57 (1)	No (< LOD)
			C1-Day 2		2020-08-26T10:57 (2)	No (< LOD)
			C1-Day 8		2020-09-01T12:00 (8)	No (< LOD)
			C1-Day 15		2020-09-08T13:45 (15)	No (< LOD)
3: 1.7E10 NET	102-079	Viremia PK (copies/ug)	Day 1	Pre-Infusion	2020-10-13T11:43 (1)	No (< LOD)
			Day 1	End of Infusion	2020-10-13T12:30 (1)	2160000 +/- 82000
			Day 1	4 Hrs Post Infusion	2020-10-13T16:30 (1)	No (< LOD)
			Day 2		2020-10-14T11:03 (2)	No (< LOD)
			Day 8		2020-10-20T10:32 (8)	No (< LOD)
			Day 15		2020-10-27T11:58 (15)	No (< LOD)

Cohorts are noted according to their study Part (1, 2, or 3) followed by their VSV-IFNß-NIS dosage in units of TCID50. Administration in Part 1 was IT monotherapy, in Part 2 was IV monotherapy, in Part 3 run-in and mCRC cohorts was IV combination therapy, and in Part 3 NET cohort was IV monotherapy.
Note: Subjects with shorter infusion durations than planned are grouped with the duration cohorts matching their actual infusion duration times and are noted with an asterisk.
Note: Results presented as mean +/- standard deviation.
The Limit of Detection (LOD) is 300 copies/ug.

Listing 16.2.10.1 VSV-IFNß-NIS Plasma Concentrations
(PK/PD Population)

Cohort	Subject Number	Test (unit)	Visit	Time Point	Date/Time (Day) YYYY-MM-DDTHH:MM	Result
3: 1.7E10 NET	104-087	Viremia PK (copies/ug)	Day 1	Pre-Infusion	2020-11-16T09:42 (1)	No (< LOD)
			Day 1	End of Infusion	2020-11-16T10:33 (1)	14600000 +/- 912000
			Day 1	4 Hrs Post Infusion	2020-11-16T14:01 (1)	292000 +/- 19100
			Day 2		2020-11-17T08:20 (2)	No (< LOD)
			Day 4		2020-11-19T13:41 (4)	No (< LOD)
			Day 8		2020-11-23T16:00 (8)	No (< LOD)
			Day 15		2020-12-01T16:00 (16)	No (< LOD)
	110-073	Viremia PK (copies/ug)	Day 1	Pre-Infusion	2020-08-18T10:05 (1)	No (< LOD)
			Day 1	End of Infusion	2020-08-18T11:00 (1)	97000 +/- 14400
			Day 1	4 Hrs Post Infusion	2020-08-18T14:56 (1)	8240 +/- 519
			Day 2		2020-08-19T13:00 (2)	No (< LOD)
			Day 8		2020-08-25T10:15 (8)	No (< LOD)
			Day 15		2020-09-01T10:30 (15)	No (< LOD)
	110-078	Viremia PK (copies/ug)	Day 1	Pre-Infusion	2020-10-01T09:03 (1)	No (< LOD)
			Day 1	End of Infusion	2020-10-01T10:16 (1)	171000 +/- 4940
			Day 1	4 Hrs Post Infusion	2020-10-01T14:15 (1)	7690 +/- 895
			Day 2		2020-10-02T11:25 (2)	No (< LOD)
			Day 8		2020-10-08T11:44 (8)	No (< LOD)
			Day 15		2020-10-15T10:15 (15)	No (< LOD)

Cohorts are noted according to their study Part (1, 2, or 3) followed by their VSV-IFNß-NIS dosage in units of TCID50. Administration in Part 1 was IT monotherapy, in Part 2 was IV monotherapy, in Part 3 run-in and mCRC cohorts was IV combination therapy, and in Part 3 NET cohort was IV monotherapy.
Note: Subjects with shorter infusion durations than planned are grouped with the duration cohorts matching their actual infusion duration times and are noted with an asterisk.
Note: Results presented as mean +/- standard deviation.
The Limit of Detection (LOD) is 300 copies/ug.
