# Supplementary material for: Phase I Study of Oncolytic Virus VV1 as Monotherapy or in Combination with Avelumab in Patients with Relapsed Refractory Solid Tumors
Source: Cancer Res Commun. 2026 Aug 5;6(8):1862–74. doi: 10.1158/2767-9764.CRC-25-0803 (PMC13440224; doi:10.1158/2767-9764.CRC-25-0803)
Supplement: Supplementary file 2 [file crc-25-0803_table_s8_suppst8.pdf]

Listing 16.2.11.1 Viral Shedding
(PK/PD Population)

				Buccal Swab		Urine Pellet		Vessicle Swab	
Cohort	Subject Number	Visit	Date/Time (Day) YYYY-MM-DDTHH:MM	IVR (TCID50/mL)	PCR (copies/sample)	IVR (TCID50/mL)	PCR (copies/sample)	IVR (TCID50/mL)	PCR (copies/sample)
1: 3E6 IT	102-001	Day 1	2017-04-26T09:40 (1)	No (< LOD)	No (< LOD)	No (< LOD)	No (< LOD)		
		Day 8	2017-05-03T08:20 (8)			No (< LOD)	No (< LOD)		
			2017-05-03T09:40 (8)	No (< LOD)	No (< LOD)				
		Day 15	2017-05-10T08:51 (15)			No (< LOD)	No (< LOD)		
			2017-05-10T09:16 (15)	No (< LOD)	No (< LOD)				
	102-002	Day 1	2017-05-03T08:30 (1)	No (< LOD)	No (< LOD)	No (< LOD)	No (< LOD)		
		Day 8	2017-05-10T08:23 (8)	No (< LOD)	No (< LOD)	No (< LOD)	No (< LOD)		
		Day 15	2017-05-17T08:31 (15)	No (< LOD)	No (< LOD)				
	102-003	Day 1	2017-06-21T08:50 (1)	No (< LOD)	No (< LOD)				
			2017-06-21T09:40 (1)			No (< LOD)	No (< LOD)		
		Day 8	2017-06-28T08:00 (8)			No (< LOD)	No (< LOD)		
			2017-06-28T08:40 (8)	No (< LOD)	No (< LOD)				
		Day 15	2017-07-06T07:45 (16)			No (< LOD)	No (< LOD)		
			2017-07-06T08:15 (16)	No (< LOD)	No (< LOD)				
1: 1E7 IT	102-004	Day 1	2017-08-09T07:50 (1)			No (< LOD)	No (< LOD)		
			2017-08-09T07:55 (1)	No (< LOD)	No (< LOD)				
		Day 8	2017-08-16T08:01 (8)			No (< LOD)	No (< LOD)		
			2017-08-16T09:01 (8)	No (< LOD)	No (< LOD)				
		Day 15	2017-08-23T07:50 (15)			No (< LOD)	No (< LOD)		

Cohorts are noted according to their study Part (1, 2, or 3) followed by their VSV-IFNß-NIS dosage in units of TCID50. Administration in Part 1 was IT monotherapy, in Part 2 was IV monotherapy, in Part 3 run-in and mCRC cohorts was IV combination therapy, and in Part 3 NET cohort was IV monotherapy.
Note: Subjects with shorter infusion durations than planned are grouped with the duration cohorts matching their actual infusion duration times and are noted with an asterisk.

Listing 16.2.11.1 Viral Shedding
(PK/PD Population)

				Buccal Swab		Urine Pellet		Vessicle Swab	
Cohort	Subject Number	Visit	Date/Time (Day) YYYY-MM-DDTHH:MM	IVR (TCID50/mL)	PCR (copies/sample)	IVR (TCID50/mL)	PCR (copies/sample)	IVR (TCID50/mL)	PCR (copies/sample)
1: 1E7 IT	102-004	Day 15	2017-08-23T08:45 (15)	No (< LOD)	No (< LOD)				
	104-007	Day 1	2017-11-01T07:02 (1)	No (< LOD)	No (< LOD)	No (< LOD)	No (< LOD)		
		Day 8	2017-11-08T07:10 (8)	No (< LOD)	No (< LOD)				
			2017-11-08T09:49 (8)			No (< LOD)	No (< LOD)		
		Day 15	2017-11-13T18:00 (13)	No (< LOD)	No (< LOD)	No (< LOD)	No (< LOD)		
	104-009	Day 1	2017-11-13T08:35 (1)	No (< LOD)	No (< LOD)	No (< LOD)	No (< LOD)		
		Day 8	2017-11-20T15:05 (8)	No (< LOD)	No (< LOD)	No (< LOD)	No (< LOD)		
		Day 15	2017-11-27T15:02 (15)	No (< LOD)	No (< LOD)	No (< LOD)	No (< LOD)		
1: 3E7 IT	103-014	Day 1	2018-03-12T07:05 (1)	No (< LOD)	No (< LOD)				
			2018-03-12T07:25 (1)			No (< LOD)	No (< LOD)		
		Day 8	2018-03-19T08:55 (8)			No (< LOD)	No (< LOD)		
			2018-03-19T09:27 (8)	No (< LOD)	No (< LOD)				
		Day 15	2018-03-26T09:00 (15)			No (< LOD)	No (< LOD)		
			2018-03-26T09:05 (15)	No (< LOD)	No (< LOD)				
	103-015	Day 1	2018-03-20T09:00 (1)			No (< LOD)	No (< LOD)		
			2018-03-20T09:06 (1)	No (< LOD)	No (< LOD)				
		Day 8	2018-03-27T12:40 (8)	No (< LOD)	No (< LOD)				
			2018-03-27T13:00 (8)			No (< LOD)	No (< LOD)		
		Day 15	2018-04-03T12:05 (15)			No (< LOD)	No (< LOD)		
			2018-04-03T12:26 (15)	No (< LOD)	No (< LOD)				

Cohorts are noted according to their study Part (1, 2, or 3) followed by their VSV-IFNß-NIS dosage in units of TCID50. Administration in Part 1 was IT monotherapy, in Part 2 was IV monotherapy, in Part 3 run-in and mCRC cohorts was IV combination therapy, and in Part 3 NET cohort was IV monotherapy.
Note: Subjects with shorter infusion durations than planned are grouped with the duration cohorts matching their actual infusion duration times and are noted with an asterisk.

Listing 16.2.11.1 Viral Shedding
(PK/PD Population)

				Buccal Swab		Urine Pellet		Vessicle Swab	
Cohort	Subject Number	Visit	Date/Time (Day) YYYY-MM-DDTHH:MM	IVR (TCID50/mL)	PCR (copies/sample)	IVR (TCID50/mL)	PCR (copies/sample)	IVR (TCID50/mL)	PCR (copies/sample)
1: 3E7 IT	105-011	Day 1	2018-01-17T09:40 (1)			No (< LOD)	No (< LOD)		
			2018-01-17T11:41 (1)	No (< LOD)	No (< LOD)				
		Day 8	2018-01-24T10:13 (8)			No (< LOD)	No (< LOD)		
			2018-01-24T12:07 (8)	No (< LOD)	No (< LOD)				
		Day 15	2018-01-31T11:38 (15)	No (< LOD)	No (< LOD)				
	105-012	Day 1	2018-02-05T10:56 (1)	No (< LOD)	No (< LOD)				
			2018-02-05T14:15 (1)			No (< LOD)	No (< LOD)		
		Day 8	2018-02-12T08:45 (8)			No (< LOD)	No (< LOD)		
			2018-02-12T09:16 (8)	No (< LOD)	No (< LOD)				
		Day 15	2018-02-19T08:14 (15)			No (< LOD)	No (< LOD)		
			2018-02-19T09:40 (15)	No (< LOD)	No (< LOD)				
1: 1E8 IT	104-017	Day 1	2018-04-16T07:15 (1)			No (< LOD)	No (< LOD)		
			2018-04-16T08:17 (1)	No (< LOD)	No (< LOD)				
		Day 8	2018-04-23T10:00 (8)	No (< LOD)	No (< LOD)	No (< LOD)	No (< LOD)		
		Day 15	2018-04-30T11:00 (15)	No (< LOD)	No (< LOD)	No (< LOD)	No (< LOD)		
	104-019	Day 1	2018-05-07T07:31 (1)	No (< LOD)	No (< LOD)				
			2018-05-07T07:36 (1)			No (< LOD)	No (< LOD)		
		Day 8	2018-05-14T09:40 (8)	No (< LOD)	No (< LOD)	No (< LOD)	No (< LOD)		
		Day 15	2018-05-21T09:29 (15)	No (< LOD)	No (< LOD)				
			2018-05-21T09:36 (15)			No (< LOD)	No (< LOD)		

Cohorts are noted according to their study Part (1, 2, or 3) followed by their VSV-IFNß-NIS dosage in units of TCID50. Administration in Part 1 was IT monotherapy, in Part 2 was IV monotherapy, in Part 3 run-in and mCRC cohorts was IV combination therapy, and in Part 3 NET cohort was IV monotherapy.
Note: Subjects with shorter infusion durations than planned are grouped with the duration cohorts matching their actual infusion duration times and are noted with an asterisk.

Listing 16.2.11.1 Viral Shedding
(PK/PD Population)

Cohort	Subject Number	Visit	Date/Time (Day) YYYY-MM-DDTHH:MM	Buccal Swab IVR (TCID50/mL)	Buccal Swab PCR (copies/sample)	Urine Pellet IVR (TCID50/mL)	Urine Pellet PCR (copies/sample)	Vessicle Swab IVR (TCID50/mL)	Vessicle Swab PCR (copies/sample)
1: 1E8 IT	105-016	Day 1	2018-04-30T11:35 (1)			No (< LOD)	No (< LOD)		
			2018-04-30T13:17 (1)	No (< LOD)	No (< LOD)				
		Day 8	2018-05-07T09:01 (8)			No (< LOD)	No (< LOD)		
			2018-05-07T09:48 (8)	No (< LOD)	No (< LOD)				
		Day 15	2018-05-14T10:26 (15)	No (< LOD)	No (< LOD)	No (< LOD)	No (< LOD)		
	105-020	Day 1	2018-05-15T11:20 (1)			No (< LOD)	No (< LOD)		
			2018-05-15T11:46 (1)	No (< LOD)	No (< LOD)				
		Day 8	2018-05-22T08:54 (8)	No (< LOD)	No (< LOD)				
			2018-05-22T09:47 (8)			No (< LOD)	No (< LOD)		
		Day 15	2018-05-30T10:35 (16)			No (< LOD)	No (< LOD)		
			2018-05-30T11:36 (16)	No (< LOD)	No (< LOD)				
1: 3E8 IT	104-023	Day 1	2018-06-27T08:35 (1)	No (< LOD)	No (< LOD)				
			2018-06-27T09:52 (1)			No (< LOD)	No (< LOD)		
		Day 8	2018-07-03T08:58 (7)	No (< LOD)	No (< LOD)	No (< LOD)	No (< LOD)		
		Day 15	2018-07-10T07:51 (14)	No (< LOD)	No (< LOD)	No (< LOD)	No (< LOD)		
	104-030	Day 1	2018-09-12T10:17 (1)	No (< LOD)	No (< LOD)				
			2018-09-12T10:47 (1)			No (< LOD)	No (< LOD)		
		Day 8	2018-09-19T12:40 (8)	No (< LOD)	No (< LOD)	No (< LOD)	No (< LOD)		
		Day 15	2018-09-26T12:35 (15)	No (< LOD)	No (< LOD)	No (< LOD)	No (< LOD)		
	105-021	Day 1	2018-06-05T11:10 (1)			No (< LOD)	No (< LOD)		

Cohorts are noted according to their study Part (1, 2, or 3) followed by their VSV-IFNß-NIS dosage in units of TCID50. Administration in Part 1 was IT monotherapy, in Part 2 was IV monotherapy, in Part 3 run-in and mCRC cohorts was IV combination therapy, and in Part 3 NET cohort was IV monotherapy.
Note: Subjects with shorter infusion durations than planned are grouped with the duration cohorts matching their actual infusion duration times and are noted with an asterisk.

Listing 16.2.11.1 Viral Shedding
(PK/PD Population)

				Buccal Swab		Urine Pellet		Vessicle Swab	
Cohort	Subject Number	Visit	Date/Time (Day) YYYY-MM-DDTHH:MM	IVR (TCID50/mL)	PCR (copies/sample)	IVR (TCID50/mL)	PCR (copies/sample)	IVR (TCID50/mL)	PCR (copies/sample)
1: 3E8 IT	105-021	Day 1	2018-06-05T11:45 (1)	No (< LOD)	No (< LOD)				
		Day 8	2018-06-12T10:05 (8)	No (< LOD)	No (< LOD)				
			2018-06-12T14:07 (8)			No (< LOD)	No (< LOD)		
		Day 15	2018-06-21T09:36 (17)	No (< LOD)	No (< LOD)				
			2018-06-21T12:03 (17)			No (< LOD)	No (< LOD)		
	106-022	Day 1	2018-06-12T09:35 (1)	No (< LOD)	No (< LOD)				
			2018-06-12T10:47 (1)			No (< LOD)	No (< LOD)		
		Day 8	2018-06-19T09:15 (8)	No (< LOD)	No (< LOD)				
			2018-06-19T10:00 (8)			No (< LOD)	No (< LOD)		
		Day 15	2018-06-25T08:42 (14)	No (< LOD)	No (< LOD)				
			2018-06-25T09:50 (14)			No (< LOD)	No (< LOD)		
1: 1E9 IT	102-028	Day 1	2018-09-12T07:10 (1)	No (< LOD)	No (< LOD)				
		Day 8	2018-09-19T08:40 (8)				No (< LOD)		
			2018-09-19T09:30 (8)	No (< LOD)	No (< LOD)				
		Day 15	2018-09-26T09:51 (15)	No (< LOD)	No (< LOD)				
			2018-09-27T05:00 (16)				No (< LOD)		
	103-025	Day 1	2018-07-31T07:15 (1)			No (< LOD)	No (< LOD)		
			2018-07-31T07:30 (1)	No (< LOD)	No (< LOD)				
		Day 8	2018-08-07T09:33 (8)	No (< LOD)	No (< LOD)				
			2018-08-07T09:35 (8)			No (< LOD)	No (< LOD)		
		Day 15	2018-08-14T09:45 (15)			No (< LOD)	No (< LOD)		

Cohorts are noted according to their study Part (1, 2, or 3) followed by their VSV-IFNß-NIS dosage in units of TCID50. Administration in Part 1 was IT monotherapy, in Part 2 was IV monotherapy, in Part 3 run-in and mCRC cohorts was IV combination therapy, and in Part 3 NET cohort was IV monotherapy.
Note: Subjects with shorter infusion durations than planned are grouped with the duration cohorts matching their actual infusion duration times and are noted with an asterisk.

Listing 16.2.11.1 Viral Shedding
(PK/PD Population)

				Buccal Swab		Urine Pellet		Vessicle Swab	
Cohort	Subject Number	Visit	Date/Time (Day) YYYY-MM-DDTHH:MM	IVR (TCID50/mL)	PCR (copies/sample)	IVR (TCID50/mL)	PCR (copies/sample)	IVR (TCID50/mL)	PCR (copies/sample)
1: 1E9 IT	103-025	Day 15	2018-08-14T10:15 (15)	No (< LOD)	No (< LOD)				
	103-029	Day 1	2018-09-05T06:59 (1)			No (< LOD)	No (< LOD)		
			2018-09-05T07:20 (1)	No (< LOD)	No (< LOD)				
		Day 8	2018-09-12T12:20 (8)			No (< LOD)	No (< LOD)		
			2018-09-12T12:44 (8)	No (< LOD)	58300 +/- 1390				
		Day 15	2018-09-19T11:50 (15)			No (< LOD)	No (< LOD)		
			2018-09-19T12:06 (15)	No (< LOD)	No (< LOD)				
	105-026	Day 1	2018-08-13T11:05 (1)			No (< LOD)	No (< LOD)		
			2018-08-13T13:03 (1)	No (< LOD)	No (< LOD)				
		Day 8	2018-08-20T10:02 (8)			No (< LOD)	No (< LOD)		
			2018-08-22T09:20 (10)	No (< LOD)	No (< LOD)				
		Day 15	2018-08-29T08:17 (17)			No (< LOD)	No (< LOD)		
			2018-08-29T09:20 (17)	No (< LOD)	No (< LOD)				
	105-027	Day 1	2018-08-20T12:00 (1)			No (< LOD)	No (< LOD)		
			2018-08-20T13:05 (1)	No (< LOD)	No (< LOD)				
		Day 8	2018-08-27T10:30 (8)			No (< LOD)	No (< LOD)		
			2018-08-27T14:45 (8)	No (< LOD)	No (< LOD)				
		Day 15	2018-09-05T09:27 (17)			No (< LOD)	No (< LOD)		
			2018-09-05T09:32 (17)	No (< LOD)	No (< LOD)				
	106-032	Day 1	2018-10-16T07:25 (1)			No (< LOD)	No (< LOD)		

Cohorts are noted according to their study Part (1, 2, or 3) followed by their VSV-IFNß-NIS dosage in units of TCID50. Administration in Part 1 was IT monotherapy, in Part 2 was IV monotherapy, in Part 3 run-in and mCRC cohorts was IV combination therapy, and in Part 3 NET cohort was IV monotherapy.
Note: Subjects with shorter infusion durations than planned are grouped with the duration cohorts matching their actual infusion duration times and are noted with an asterisk.

Listing 16.2.11.1 Viral Shedding
(PK/PD Population)

				Buccal Swab		Urine Pellet		Vessicle Swab	
Cohort	Subject Number	Visit	Date/Time (Day) YYYY-MM-DDTHH:MM	IVR (TCID50/mL)	PCR (copies/sample)	IVR (TCID50/mL)	PCR (copies/sample)	IVR (TCID50/mL)	PCR (copies/sample)
1: 1E9 IT	106-032	Day 1	2018-10-16T08:30 (1)	No (< LOD)	No (< LOD)				
		Day 8	2018-10-23T10:07 (8)	No (< LOD)	No (< LOD)				
			2018-10-23T10:25 (8)			No (< LOD)	No (< LOD)		
		Day 15	2018-10-29T10:50 (14)	No (< LOD)	No (< LOD)				
			2018-10-29T11:07 (14)			No (< LOD)	No (< LOD)		
1: 3E9 IT	102-033	Day 1	2018-11-19T07:52 (1)	No (< LOD)	No (< LOD)				
			2018-11-19T08:19 (1)			No (< LOD)	No (< LOD)		
		Day 8	2018-11-26T09:45 (8)			No (< LOD)	No (< LOD)		
			2018-11-26T10:42 (8)	No (< LOD)	No (< LOD)				
		Day 15	2018-12-03T09:26 (15)			No (< LOD)	No (< LOD)		
			2018-12-03T10:20 (15)	No (< LOD)	No (< LOD)				
	104-039	Day 1	2019-01-02T08:30 (1)			No (< LOD)	No (< LOD)		
			2019-01-02T09:00 (1)	No (< LOD)	No (< LOD)				
		Day 8	2019-01-09T17:40 (8)	No (< LOD)	No (< LOD)	No (< LOD)	No (< LOD)		
		Day 15	2019-01-17T15:28 (16)	No (< LOD)	No (< LOD)	No (< LOD)	No (< LOD)		
	105-038	Day 1	2018-12-03T11:10 (1)			No (< LOD)	No (< LOD)		
			2018-12-03T12:56 (1)	No (< LOD)	No (< LOD)				
		Day 8	2018-12-10T09:35 (8)	No (< LOD)	No (< LOD)				
			2018-12-10T10:08 (8)			No (< LOD)	No (< LOD)		
		Day 15	2018-12-19T08:47 (17)			No (< LOD)	No (< LOD)		

Cohorts are noted according to their study Part (1, 2, or 3) followed by their VSV-IFNß-NIS dosage in units of TCID50. Administration in Part 1 was IT monotherapy, in Part 2 was IV monotherapy, in Part 3 run-in and mCRC cohorts was IV combination therapy, and in Part 3 NET cohort was IV monotherapy.
Note: Subjects with shorter infusion durations than planned are grouped with the duration cohorts matching their actual infusion duration times and are noted with an asterisk.

Listing 16.2.11.1 Viral Shedding
(PK/PD Population)

				Buccal Swab		Urine Pellet		Vessicle Swab	
Cohort	Subject Number	Visit	Date/Time (Day) YYYY-MM-DDTHH:MM	IVR (TCID50/mL)	PCR (copies/sample)	IVR (TCID50/mL)	PCR (copies/sample)	IVR (TCID50/mL)	PCR (copies/sample)
1: 3E9 IT	105-038	Day 15	2018-12-19T10:00 (17)	No (< LOD)	No (< LOD)				
2: 1.7E10 15m	102-061*	Day 1	2020-01-21T08:33 (1)	No (< LOD)	No (< LOD)				
		Day 8	2020-01-28T08:45 (8)	No (< LOD)	No (< LOD)				
		Day 15	2020-02-06T09:30 (17)	No (< LOD)	No (< LOD)				
	102-062	Day 1	2020-01-22T08:02 (1)	No (< LOD)	No (< LOD)				
		Day 8	2020-01-29T08:35 (8)	No (< LOD)	No (< LOD)				
		Day 15	2020-02-04T09:01 (14)	No (< LOD)	No (< LOD)				
	103-070	Day 1	2020-06-09T10:45 (1)	No (< LOD)	No (< LOD)				
		Day 8	2020-06-16T08:30 (8)	No (< LOD)	No (< LOD)				
	104-067	Day 1	2020-03-24T10:45 (1)	No (< LOD)	No (< LOD)				
		Day 8	2020-03-31T12:52 (8)	No (< LOD)	No (< LOD)				
		Day 15	2020-04-07T13:11 (15)	No (< LOD)	No (< LOD)				
	105-063	Day 1	2020-01-29T15:12 (1)	No (< LOD)	No (< LOD)				
		Day 8	2020-02-05T09:30 (8)	No (< LOD)	No (< LOD)				
		Day 15	2020-02-12T09:50 (15)	No (< LOD)	No (< LOD)				
2: 1.7E10 30m	102-044	Day 1	2019-04-08T10:00 (1)	No (< LOD)	No (< LOD)				
		Day 8	2019-04-15T09:37 (8)	No (< LOD)	No (< LOD)				
		Day 15	2019-04-22T10:54 (15)	No (< LOD)	No (< LOD)				

Cohorts are noted according to their study Part (1, 2, or 3) followed by their VSV-IFNß-NIS dosage in units of TCID50. Administration in Part 1 was IT monotherapy, in Part 2 was IV monotherapy, in Part 3 run-in and mCRC cohorts was IV combination therapy, and in Part 3 NET cohort was IV monotherapy.
Note: Subjects with shorter infusion durations than planned are grouped with the duration cohorts matching their actual infusion duration times and are noted with an asterisk.

Listing 16.2.11.1 Viral Shedding
(PK/PD Population)

				Buccal Swab		Urine Pellet		Vessicle Swab	
Cohort	Subject Number	Visit	Date/Time (Day) YYYY-MM-DDTHH:MM	IVR (TCID50/mL)	PCR (copies/sample)	IVR (TCID50/mL)	PCR (copies/sample)	IVR (TCID50/mL)	PCR (copies/sample)
2: 1.7E10 30m	103-046	Day 1	2019-04-15T07:35 (1)	No (< LOD)	No (< LOD)				
		Day 8	2019-04-22T11:44 (8)	No (< LOD)	No (< LOD)				
		Day 15	2019-04-29T10:47 (15)	No (< LOD)	No (< LOD)				
	103-060*	Day 1	2019-07-31T08:39 (1)	No (< LOD)	No (< LOD)				
		Day 8	2019-08-07T11:35 (8)	No (< LOD)	No (< LOD)				
		Day 15	2019-08-14T11:18 (15)	No (< LOD)	No (< LOD)				
	105-040	Day 1	2019-02-11T10:55 (1)	No (< LOD)	No (< LOD)				
			2019-02-11T11:30 (1)			No (< LOD)			
		Day 8	2019-02-18T10:00 (8)			No (< LOD)			
			2019-02-18T10:20 (8)	No (< LOD)	No (< LOD)				
		Day 15	2019-02-27T14:37 (17)			No (< LOD)			
			2019-02-27T15:13 (17)	No (< LOD)	No (< LOD)				
	105-041	Day 1	2019-02-18T13:03 (1)	No (< LOD)	No (< LOD)				
		Day 8	2019-02-25T10:36 (8)	No (< LOD)	No (< LOD)				
		Day 15	2019-03-06T10:48 (17)	No (< LOD)	No (< LOD)				
			2019-03-06T11:00 (17)					No (< LOD)	
	105-042	Day 1	2019-03-18T10:15 (1)	No (< LOD)	No (< LOD)				
		Day 8	2019-03-25T10:06 (8)	No (< LOD)	No (< LOD)				
		Day 15	2019-04-03T10:15 (17)	No (< LOD)	No (< LOD)				

Cohorts are noted according to their study Part (1, 2, or 3) followed by their VSV-IFNß-NIS dosage in units of TCID50. Administration in Part 1 was IT monotherapy, in Part 2 was IV monotherapy, in Part 3 run-in and mCRC cohorts was IV combination therapy, and in Part 3 NET cohort was IV monotherapy.
Note: Subjects with shorter infusion durations than planned are grouped with the duration cohorts matching their actual infusion duration times and are noted with an asterisk.

Listing 16.2.11.1 Viral Shedding
(PK/PD Population)

				Buccal Swab		Urine Pellet		Vessicle Swab	
Cohort	Subject Number	Visit	Date/Time (Day) YYYY-MM-DDTHH:MM	IVR (TCID50/mL)	PCR (copies/sample)	IVR (TCID50/mL)	PCR (copies/sample)	IVR (TCID50/mL)	PCR (copies/sample)
2: 1.7E10 30m	106-043	Day 1	2019-04-08T10:15 (1)	No (< LOD)	No (< LOD)				
		Day 8	2019-04-15T09:00 (8)	No (< LOD)	No (< LOD)				
		Day 15	2019-04-22T10:50 (15)	No (< LOD)	No (< LOD)				
2: 1.7E10 60m	102-057	Day 1	2019-07-01T09:16 (1)	No (< LOD)	No (< LOD)				
		Day 8	2019-07-08T09:32 (8)	No (< LOD)	No (< LOD)				
		Day 15	2019-07-15T11:17 (15)	No (< LOD)	No (< LOD)				
	102-058	Day 1	2019-07-01T08:45 (1)	No (< LOD)	No (< LOD)				
		Day 8	2019-07-08T08:50 (8)	No (< LOD)	No (< LOD)				
		Day 15	2019-07-15T11:04 (15)	No (< LOD)	No (< LOD)				
	104-056	Day 1	2019-06-19T09:45 (1)	No (< LOD)	No (< LOD)				
		Day 8	2019-06-26T09:20 (8)	No (< LOD)	No (< LOD)				
		Day 15	2019-07-03T14:05 (15)	No (< LOD)	No (< LOD)				
	105-055	Day 1	2019-06-10T09:24 (1)	No (< LOD)	No (< LOD)				
		Day 8	2019-06-17T09:28 (8)	No (< LOD)	No (< LOD)				
		Day 15	2019-06-26T09:46 (17)	No (< LOD)	No (< LOD)				
	105-059	Day 1	2019-07-15T11:19 (1)	No (< LOD)	No (< LOD)				
		Day 8	2019-07-22T12:18 (8)	No (< LOD)	No (< LOD)				
		Day 15	2019-07-31T12:08 (17)	No (< LOD)	No (< LOD)				

Cohorts are noted according to their study Part (1, 2, or 3) followed by their VSV-IFNß-NIS dosage in units of TCID50. Administration in Part 1 was IT monotherapy, in Part 2 was IV monotherapy, in Part 3 run-in and mCRC cohorts was IV combination therapy, and in Part 3 NET cohort was IV monotherapy.
Note: Subjects with shorter infusion durations than planned are grouped with the duration cohorts matching their actual infusion duration times and are noted with an asterisk.

Listing 16.2.11.1 Viral Shedding
(PK/PD Population)

				Buccal Swab		Urine Pellet		Vessicle Swab	
Cohort	Subject Number	Visit	Date/Time (Day) YYYY-MM-DDTHH:MM	IVR (TCID50/mL)	PCR (copies/sample)	IVR (TCID50/mL)	PCR (copies/sample)	IVR (TCID50/mL)	PCR (copies/sample)
2: 1.7E10 180m	102-048	Day 1	2019-04-29T09:02 (1)	No (< LOD)	No (< LOD)				
		Day 8	2019-05-06T14:00 (8)	No (< LOD)	No (< LOD)				
		Day 15	2019-05-14T15:20 (16)	No (< LOD)	No (< LOD)				
	102-053	Day 1	2019-05-14T10:20 (1)	No (< LOD)	No (< LOD)				
		Day 8	2019-05-21T10:46 (8)	No (< LOD)	No (< LOD)				
		Day 15	2019-05-30T10:19 (17)	No (< LOD)	No (< LOD)				
	102-054	Day 1	2019-05-29T08:14 (1)	No (< LOD)	No (< LOD)				
		Day 8	2019-06-05T10:21 (8)	No (< LOD)	No (< LOD)				
		Day 15	2019-06-13T10:00 (16)	No (< LOD)	No (< LOD)				
	104-047	Day 1	2019-04-23T08:24 (1)	No (< LOD)	No (< LOD)				
		Day 8	2019-04-30T10:51 (8)	No (< LOD)	No (< LOD)				
		Day 15	2019-05-07T09:47 (15)	No (< LOD)	No (< LOD)				
	104-050	Day 1	2019-05-01T10:27 (1)	No (< LOD)	No (< LOD)				
		Day 8	2019-05-08T16:05 (8)	No (< LOD)	No (< LOD)				
		Day 15	2019-05-15T14:15 (15)	No (< LOD)	No (< LOD)				
	105-052	Day 1	2019-05-28T09:42 (1)	No (< LOD)	No (< LOD)				
		Day 8	2019-06-04T11:24 (8)	No (< LOD)	No (< LOD)				
		Day 15	2019-06-12T13:06 (16)	No (< LOD)	No (< LOD)				

Cohorts are noted according to their study Part (1, 2, or 3) followed by their VSV-IFNß-NIS dosage in units of TCID50. Administration in Part 1 was IT monotherapy, in Part 2 was IV monotherapy, in Part 3 run-in and mCRC cohorts was IV combination therapy, and in Part 3 NET cohort was IV monotherapy.
Note: Subjects with shorter infusion durations than planned are grouped with the duration cohorts matching their actual infusion duration times and are noted with an asterisk.

Listing 16.2.11.1 Viral Shedding
(PK/PD Population)

				Buccal Swab		Urine Pellet		Vessicle Swab	
Cohort	Subject Number	Visit	Date/Time (Day) YYYY-MM-DDTHH:MM	IVR (TCID50/mL)	PCR (copies/sample)	IVR (TCID50/mL)	PCR (copies/sample)	IVR (TCID50/mL)	PCR (copies/sample)
2: 1.0E11 MTD	102-093	Day 1	2021-04-26T08:40 (1)	No (< LOD)	No (< LOD)				
		Day 8	2021-05-03T12:00 (8)	No (< LOD)	No (< LOD)				
		Day 15	2021-05-10T10:15 (15)	No (< LOD)	No (< LOD)				
	104-095	Day 1	2021-04-26T09:05 (1)	No (< LOD)	No (< LOD)				
		Day 8	2021-05-03T13:55 (8)	No (< LOD)					
		Day 15	2021-05-12T10:00 (17)	No (< LOD)					
		Day 22	2021-05-19T10:50 (24)		No (< LOD)				
	104-096	Day 1	2021-05-04T10:40 (1)	No (< LOD)	No (< LOD)				
		Day 8	2021-05-11T13:19 (8)	No (< LOD)	No (< LOD)				
		Day 15	2021-05-19T09:05 (16)	No (< LOD)	No (< LOD)				
	104-097	Day 1	2021-05-18T10:50 (1)	No (< LOD)	No (< LOD)				
		Day 8	2021-05-25T12:30 (8)	No (< LOD)	No (< LOD)				
		Day 15	2021-06-02T10:51 (16)	No (< LOD)	No (< LOD)				
	104-098	Day 1	2021-05-10T09:20 (1)	No (< LOD)	No (< LOD)				
		Day 8	2021-05-17T13:55 (8)	No (< LOD)	No (< LOD)				
		Day 15	2021-05-26T11:08 (17)	No (< LOD)	No (< LOD)				
	104-099	Day 1	2021-06-01T11:16 (1)	No (< LOD)	No (< LOD)				
		Day 8	2021-06-08T13:20 (8)	No (< LOD)	No (< LOD)				

Cohorts are noted according to their study Part (1, 2, or 3) followed by their VSV-IFNß-NIS dosage in units of TCID50. Administration in Part 1 was IT monotherapy, in Part 2 was IV monotherapy, in Part 3 run-in and mCRC cohorts was IV combination therapy, and in Part 3 NET cohort was IV monotherapy.
Note: Subjects with shorter infusion durations than planned are grouped with the duration cohorts matching their actual infusion duration times and are noted with an asterisk.

Listing 16.2.11.1 Viral Shedding
(PK/PD Population)

				Buccal Swab		Urine Pellet		Vessicle Swab	
Cohort	Subject Number	Visit	Date/Time (Day) YYYY-MM-DDTHH:MM	IVR (TCID50/mL)	PCR (copies/sample)	IVR (TCID50/mL)	PCR (copies/sample)	IVR (TCID50/mL)	PCR (copies/sample)
2: 1.0E11 MTD	104-099	Day 15	2021-06-16T09:19 (16)	No (< LOD)	No (< LOD)				
3: 1.7E10 Run-In	104-068	C1-Day 1	2020-03-23T09:52 (1)	No (< LOD)	No (< LOD)				
		C1-Day 8	2020-03-30T10:02 (8)	No (< LOD)	No (< LOD)				
		C1-Day 15	2020-04-06T12:25 (15)	No (< LOD)	No (< LOD)				
	105-065	C1-Day 1	2020-02-18T10:05 (1)	No (< LOD)	No (< LOD)				
		C1-Day 8	2020-02-25T11:10 (8)	No (< LOD)	No (< LOD)				
		C1-Day 15	2020-03-05T10:40 (17)	No (< LOD)	No (< LOD)				
	105-066	C1-Day 1	2020-02-26T11:05 (1)	No (< LOD)	No (< LOD)				
		C1-Day 8	2020-03-04T13:33 (8)	No (< LOD)					
		C1-Day 15	2020-03-11T15:21 (15)	No (< LOD)	No (< LOD)				
3: 1.7E10 mCRC	102-071	C1-Day 1	2020-07-28T10:06 (1)	No (< LOD)	No (< LOD)				
		C1-Day 8	2020-08-04T09:15 (8)	No (< LOD)	No (< LOD)				
		C1-Day 15	2020-08-10T10:05 (14)	No (< LOD)	No (< LOD)				
	102-080	C1-Day 1	2020-10-20T10:21 (1)	No (< LOD)	No (< LOD)				
		C1-Day 8	2020-10-27T13:11 (8)	No (< LOD)	No (< LOD)				
		C1-Day 15	2020-11-02T11:16 (14)	No (< LOD)	No (< LOD)				
	103-076	C1-Day 1	2020-09-21T08:23 (1)	No (< LOD)	No (< LOD)				
		C1-Day 8	2020-09-30T13:00 (10)	No (< LOD)	No (< LOD)				

Cohorts are noted according to their study Part (1, 2, or 3) followed by their VSV-IFNß-NIS dosage in units of TCID50. Administration in Part 1 was IT monotherapy, in Part 2 was IV monotherapy, in Part 3 run-in and mCRC cohorts was IV combination therapy, and in Part 3 NET cohort was IV monotherapy.
Note: Subjects with shorter infusion durations than planned are grouped with the duration cohorts matching their actual infusion duration times and are noted with an asterisk.

Listing 16.2.11.1 Viral Shedding
(PK/PD Population)

				Buccal Swab		Urine Pellet		Vessicle Swab	
Cohort	Subject Number	Visit	Date/Time (Day) YYYY-MM-DDTHH:MM	IVR (TCID50/mL)	PCR (copies/sample)	IVR (TCID50/mL)	PCR (copies/sample)	IVR (TCID50/mL)	PCR (copies/sample)
3: 1.7E10 mCRC	103-076	C1-Day 15	2020-10-05T12:01 (15)	No (< LOD)	No (< LOD)				
	103-081	C1-Day 1	2020-10-12T07:41 (1)	No (< LOD)	No (< LOD)				
		C1-Day 8	2020-10-19T13:19 (8)	No (< LOD)	No (< LOD)				
		C1-Day 15	2020-10-26T12:32 (15)	No (< LOD)	No (< LOD)				
	103-082	C1-Day 1	2020-10-19T09:04 (1)	No (< LOD)	No (< LOD)				
		C1-Day 8	2020-10-26T10:54 (8)	No (< LOD)	No (< LOD)				
		C1-Day 15	2020-11-02T10:37 (15)	No (< LOD)	No (< LOD)				
	103-084	C1-Day 1	2020-11-16T07:45 (1)	No (< LOD)	No (< LOD)				
		C1-Day 8	2020-11-23T13:20 (8)	No (< LOD)	No (< LOD)				
		C1-Day 15	2020-11-30T11:37 (15)	No (< LOD)	No (< LOD)				
	103-086	C1-Day 1	2020-11-09T07:50 (1)	No (< LOD)	No (< LOD)				
		C1-Day 8	2020-11-16T11:32 (8)	No (< LOD)	No (< LOD)				
		C1-Day 15	2020-11-23T11:20 (15)	No (< LOD)	No (< LOD)				
		Unscheduled 103.1	2020-11-13T10:21 (5)		No (< LOD)			No (< LOD)	No (< LOD)
	103-088	C1-Day 1	2020-11-17T07:31 (1)	No (< LOD)	No (< LOD)				
		C1-Day 8	2020-11-24T12:52 (8)	No (< LOD)	No (< LOD)				
		C1-Day 15	2020-12-01T12:42 (15)	No (< LOD)	No (< LOD)				
	104-089	C1-Day 1	2021-01-12T10:35 (1)	No (< LOD)	No (< LOD)				
		C1-Day 8	2021-01-19T07:55 (8)	No (< LOD)	No (< LOD)				

Cohorts are noted according to their study Part (1, 2, or 3) followed by their VSV-IFNß-NIS dosage in units of TCID50. Administration in Part 1 was IT monotherapy, in Part 2 was IV monotherapy, in Part 3 run-in and mCRC cohorts was IV combination therapy, and in Part 3 NET cohort was IV monotherapy.
Note: Subjects with shorter infusion durations than planned are grouped with the duration cohorts matching their actual infusion duration times and are noted with an asterisk.

Listing 16.2.11.1 Viral Shedding
(PK/PD Population)

Cohort	Subject Number	Visit	Date/Time (Day) YYYY-MM-DDTHH:MM	Buccal Swab IVR (TCID50/mL)	Buccal Swab PCR (copies/sample)	Urine Pellet IVR (TCID50/mL)	Urine Pellet PCR (copies/sample)	Vessicle Swab IVR (TCID50/mL)	Vessicle Swab PCR (copies/sample)
3: 1.7E10 mCRC	104-089	C1-Day 15	2021-01-26T07:30 (15)	No (< LOD)	No (< LOD)				
	104-090	C1-Day 1	2021-01-19T13:25 (1)	No (< LOD)	No (< LOD)				
		C1-Day 8	2021-01-26T13:05 (8)	No (< LOD)	No (< LOD)				
		C1-Day 15	2021-02-02T07:57 (15)	No (< LOD)	No (< LOD)				
	105-085	C1-Day 1	2020-11-04T09:26 (1)	No (< LOD)	No (< LOD)				
		C1-Day 8	2020-11-11T09:10 (8)	No (< LOD)	No (< LOD)				
		C1-Day 15	2020-11-18T10:50 (15)	No (< LOD)	No (< LOD)				
	110-072	C1-Day 1	2020-08-26T09:22 (1)	No (< LOD)	No (< LOD)				
		C1-Day 8	2020-09-02T12:56 (8)	No (< LOD)	No (< LOD)				
	110-075	C1-Day 1	2020-08-25T10:00 (1)	No (< LOD)	No (< LOD)				
		C1-Day 8	2020-09-01T12:02 (8)	No (< LOD)	No (< LOD)				
		C1-Day 15	2020-09-08T13:43 (15)	No (< LOD)	No (< LOD)				
3: 1.7E10 NET	102-079	Day 1	2020-10-13T11:41 (1)	No (< LOD)	No (< LOD)				
		Day 8	2020-10-20T12:05 (8)	No (< LOD)	No (< LOD)				
		Day 15	2020-10-27T13:06 (15)	No (< LOD)	No (< LOD)				
	104-087	Day 1	2020-11-16T09:51 (1)	No (< LOD)	No (< LOD)				
		Day 8	2020-11-23T16:00 (8)	No (< LOD)	No (< LOD)				

Cohorts are noted according to their study Part (1, 2, or 3) followed by their VSV-IFNß-NIS dosage in units of TCID50. Administration in Part 1 was IT monotherapy, in Part 2 was IV monotherapy, in Part 3 run-in and mCRC cohorts was IV combination therapy, and in Part 3 NET cohort was IV monotherapy.
Note: Subjects with shorter infusion durations than planned are grouped with the duration cohorts matching their actual infusion duration times and are noted with an asterisk.

Listing 16.2.11.1 Viral Shedding
(PK/PD Population)

				Buccal Swab		Urine Pellet		Vessicle Swab	
Cohort	Subject Number	Visit	Date/Time (Day) YYYY-MM-DDTHH:MM	IVR (TCID50/mL)	PCR (copies/sample)	IVR (TCID50/mL)	PCR (copies/sample)	IVR (TCID50/mL)	PCR (copies/sample)
3: 1.7E10 NET	104-087	Day 15	2020-12-01T16:00 (16)	No (< LOD)	No (< LOD)				
	110-073	Day 1	2020-08-18T10:08 (1)	No (< LOD)	No (< LOD)				
		Day 8	2020-08-25T10:15 (8)	No (< LOD)	No (< LOD)				
		Day 15	2020-09-01T10:30 (15)	No (< LOD)	No (< LOD)				
	110-078	Day 1	2020-10-01T09:02 (1)	No (< LOD)					
		Day 8	2020-10-08T11:35 (8)	No (< LOD)					
		Day 15	2020-10-15T10:15 (15)	No (< LOD)					

Cohorts are noted according to their study Part (1, 2, or 3) followed by their VSV-IFNß-NIS dosage in units of TCID50. Administration in Part 1 was IT monotherapy, in Part 2 was IV monotherapy, in Part 3 run-in and mCRC cohorts was IV combination therapy, and in Part 3 NET cohort was IV monotherapy.
Note: Subjects with shorter infusion durations than planned are grouped with the duration cohorts matching their actual infusion duration times and are noted with an asterisk.
