# Supplementary material for: Phase I Study of Oncolytic Virus VV1 as Monotherapy or in Combination with Avelumab in Patients with Relapsed Refractory Solid Tumors
Source: Cancer Res Commun. 2026 Aug 5;6(8):1862–74. doi: 10.1158/2767-9764.CRC-25-0803 (PMC13440224; doi:10.1158/2767-9764.CRC-25-0803)
Supplement: Supplementary file 3 [file crc-25-0803_table_s10_suppst10.docx]

Table S10: Part 1 disease response and SPECT/CT positivity

| **VV1 IT dose (TCID_50_)** | **Patient no** | **Cancer type** | **Investigator response assessment  (Day 43)*** | **Positive SPEC/CT locations  (latest Day)**** | |
| --- | --- | --- | --- | --- | --- |
|  |  |  |  | **Investigator assessment** | **Central reader assessment** |
| **3 x 10^6^** | 1 | SCC | PD | None | N/A |
|  | 2 | SCC | PD | None | N/A |
|  | 3 | Adenocarcinoma | PD | Lung (Day 3) | N/A |
| **1 x 10^7^** | 4 | Adenocarcinoma | N/A | None | N/A |
|  | 5 | Adenocarcinoma | N/A | None | None |
|  | 6 | SCC | PD | None | None |
| **3 x 10^7^** | 7 | Ductal carcinoma | SD | N/A | N/A |
|  | 8 | Ductal carcinoma | PD | N/A | N/A |
|  | 9 | Adenocarcinoma | PD | Adrenal (Day 3) | None |
|  | 10 | Adenocarcinoma | PD | Other: right lateral chest wall (Day 31)^a†^ | Other: chest (Day 31)^†^ |
| **1 x 10^8^** | 11 | Other: adenoid  cystic carcinoma | SD | None | None |
|  | 12 | Adenocarcinoma | SD | None | None |
|  | 13 | SCC | SD | Other: left supraclavicular lymph node (Day 31) | Lymph node (Day 15)^b^ |
|  | 14 | Other: mesothelioma | PD | Other: right chest wall (Day 8) | Other: chest (Day 8)^c^ |
| **3 x 10^8^** | 15 | Adenocarcinoma | N/A | None | None |
|  | 16 | Adenocarcinoma | PD | Liver (Day 3) | None |
|  | 17 | Adenocarcinoma | SD | Lymph node (Day 31) | Lymph node (Day 17)^d^ |
|  | 18 | SCC | PD | Lymph node (Day 8)^e^ | Other: left cervical paraspinal mass (Day 8)^e^ |
| **1 x 10^9^** | 19 | Other: poorly differentiated serous carcinoma | N/A | N/A | N/A |
|  | 20 | Adenocarcinoma | SD | None^f^ | None^f^ |
|  | 21 | Clear cell carcinoma | SD | Other: right anterior pelvis; retroperitoneal lymphadenopathy (Day 3) | None |
|  | 22 | Adenocarcinoma | PD | None | None |
|  | 23 | Adenocarcinoma | SD | Other: left upper quadrant extending into the adjacent abdominal wall (Day 31) | Other: omentum left upper quadrant (Day 31) |
|  | 24 | Adenocarcinoma | SD | None^g^ | None |
| **3 x 10^9^** | 25 | Other: melanoma | PD | N/A | N/A |
|  | 26 | SCC | PD | None | None |
|  | 27 | Clear cell carcinoma | PD | Other: left lower anterior abdominal wall (Day 31)^h^ | Colon, Other: LLQ abdominal wall, omentum (Day 29)^h^ |

N/A = not assessed, PD = progressive disease, SCC= squamous cell carcinoma, SD = stable disease.
* Days stated are planned assessment days (range: Day 38 - Day 45).
** Days stated are actual assessment days.
† Positives observed at Day 57 (unscheduled assessment) by investigator and central reader.
Positives observed at screening:
a. Day -11 by investigator.
b. Day -14 by central reader.
c. Day -8 by central reader.
d. Day -11 by central reader.
e. Day -5 by investigator and central reader.
f. Day -6 by investigator and central reader.
g. Day -12 by investigator.
h. Day -5 by investigator and central reader.
