# Supplementary material for: Phase I Study of Oncolytic Virus VV1 as Monotherapy or in Combination with Avelumab in Patients with Relapsed Refractory Solid Tumors
Source: Cancer Res Commun. 2026 Aug 5;6(8):1862–74. doi: 10.1158/2767-9764.CRC-25-0803 (PMC13440224; doi:10.1158/2767-9764.CRC-25-0803)
Supplement: Supplementary file 4 [file crc-25-0803_table_s11_suppst11.docx]

Table S11: Study population representativeness

| **Cancer type(s)/subtype(s)/stage(s)/condition** | **Solid tumors** |
| --- | --- |
| Considerations related to: |  |
| Sex | While there are differences between the types of solid tumor that can affect men and women (e.g. breast, prostate) the overall global incidence of solid tumors is approximately equal for both sexes (9,564,954 cases among men and 9,089,887 among women).^1^  Across all 3 study parts the representation of sexes was representative. |
| Age | The median age of a cancer diagnosis in the USA is 67 years.^2^  The median age at study entry already was biased towards a significantly younger study population. |
| Race/ethnicity | In the USA from 2017-2021, the overall incidences of solid tumor cancers in black men and women were 1.1 to 191.5 per 100,000 and 0.1 to 131.3, per 100,000, respectively.^3^ In comparison, the overall incidences of solid tumor cancers among white men and women were 0.3 to 114.5, per 100,000 and 3.5 to 137.9 per 100,000.^3^  The vast majority of patients were white, non-Hispanic or Latino. |
| Geography | Of the 13 most common cancers in the USA (excluding NMSC), 11 are solid cancers.^4^ These are bladder, breast, colon/rectal, endometrial, kidney, liver and intrahepatic bile duct, lung, melanoma, pancreatic, prostate, thyroid.^4^ In 2025, it is estimated that these 11 solid tumor cancers were responsible for in 39,4150 deaths.^4^  The cancers of the study population were not representative of the most common cancers in the USA. |
| Other considerations | Based on the experience of the authors, the study population of this phase 1 trial was typical of similar trials conducted in the USA.^5^ |
| Overall representativeness of the study | This open label non-randomized study had no primary efficacy endpoint and was not meant to guide treatment. The phase 1 design this all-comer study means the population was not representative for patient care. |

**References:**

1. Bray F, Laversanne M, Sung H, Ferlay J, Siegel RL, Soerjomataram I, Jemal A. Global cancer statistics 2022: GLOBOCAN estimates of incidence and mortality worldwide for 36 cancers in 185 countries. CA Cancer J Clin. 2024;74(3):229-63. [PubMed:38572751].
2. National Cancer Institute 2025. Available at: [Risk Factors: Age - NCI](https://www.cancer.gov/about-cancer/causes-prevention/risk/age) Accessed 04.10.26.
3. Saka AH, Giaquinto AN, McCullough LE, Tossas KY, Star J, Jemal A, Siegel RL. Cancer statistics for African American and Black people, 2025. CA Cancer J Clin. 2025 ;75(2):111-40. [PubMed: 39976243].
4. National Cancer Institute 2025. Available at: [Common Cancer Types - NCI](https://www.cancer.gov/types/common-cancers) Accessed 04.10.26.
5. Farooq M, Sharon E, Takebe N. Demographic Trends in NCI-sponsored Early-phase Clinical Trials (2000-2023): A Cohort Study. Cancer Discov. 2025;15(11):2251-61. [PubMed: 40767550].
