# Supplementary material for: Phase I Study of Oncolytic Virus VV1 as Monotherapy or in Combination with Avelumab in Patients with Relapsed Refractory Solid Tumors
Source: Cancer Res Commun. 2026 Aug 5;6(8):1862–74. doi: 10.1158/2767-9764.CRC-25-0803 (PMC13440224; doi:10.1158/2767-9764.CRC-25-0803)
Supplement: Supplementary file 5 [file crc-25-0803_table_s1_suppst1.docx]

Table S1: Injection volume, site size and image guidance criteria

| **Injection site** | **Size criteria for injection^1^** | **Image guidance required** |
| --- | --- | --- |

| Skin | ≥ 1 cm | Photographs pre-injection^2^ |
| --- | --- | --- |
| Soft tissue (not penetrating skin surface and not nodal) | ≥ 1 cm | Ultrasound or CT |
| Nodal | ≥ 1.5 cm^3^ | Ultrasound or CT |
| Lung | ≥ 2 cm | CT |
| Liver (Group B only) | ≥ 1 cm and ≤ 4 cm | CT |

1. Measured in the longest dimension.
2. Photograph should include measuring tape documenting longest measurable dimension.
3. Nodal measured 1.5 cm in the shortest dimension.

**Volume of injection** (𝑽i) = (𝒂^𝟐^)(𝒃)(𝟎.𝟓)

**[a = the shorter diameter and b = the longer diameter] of injectable product.**

The volume delivered to the tumor was dependent on the size of the tumor nodule(s) and was determined according to the following algorithm:

- Up to 0.5 mL for tumors of 1.0 to 1.5 cm longest dimension.
- Up to 1.0 mL for tumors of 1.5 to 2.5 cm longest dimension*.*
- Up to 2.0 mL for tumors of 2.5 to 5 cm longest dimension.
- Up to 4.0 mL for tumors >5 cm longest diameter.
