# Supplementary material for: Phase I Study of Oncolytic Virus VV1 as Monotherapy or in Combination with Avelumab in Patients with Relapsed Refractory Solid Tumors
Source: Cancer Res Commun. 2026 Aug 5;6(8):1862–74. doi: 10.1158/2767-9764.CRC-25-0803 (PMC13440224; doi:10.1158/2767-9764.CRC-25-0803)
Supplement: Supplementary file 6 [file crc-25-0803_table_s2_suppst2.docx]

Table S2: DLT definition VV1 monotherapy

| Type | DLT Criteria |
| --- | --- |
| Non-hematologic | • Any grade ≥3 non-hematologic adverse event.  • Grade 2 or greater autoimmune disorder with the exception of vitiligo. |
| Hematologic | • Grade ≥ 3 thrombocytopenia, WBC, neutropenia, lymphopenia of 7 days’ duration or greater  • Any other Grade 4 or greater hematologic toxicity. |
| Life-threatening VSV infection | • Grade 4 VSV infection lasting 6 days or more. |
| Increased viremia | • >10-fold increase in the copy number (copies/μg RNA) between sequential samples at least 3 days apart beyond day 14. |
| Hepatic | • Isolated transaminases (AST and ALT) ≥ Grade 3 CTCAE v4.03 (>5.0 x ULN) for ≤ 6 days as long as bilirubin is ≤ 1.5 x ULN.  • Bilirubin > 3 x ULN for ≤ 6 days if isolated OR > 1.5 if accompanied by transaminase elevation ≥ Grade 3. |
