# Supplementary material for: Phase I Study of Oncolytic Virus VV1 as Monotherapy or in Combination with Avelumab in Patients with Relapsed Refractory Solid Tumors
Source: Cancer Res Commun. 2026 Aug 5;6(8):1862–74. doi: 10.1158/2767-9764.CRC-25-0803 (PMC13440224; doi:10.1158/2767-9764.CRC-25-0803)
Supplement: Supplementary file 7 [file crc-25-0803_table_s3_suppst3.docx]

Table S3: DLT definition VV1 combination therapy

| Type | DLT Criteria |
| --- | --- |
| Non-hematologic | • Any Grade ≥ 3 non-hematological treatment-emergent AE assessed as related to the combination of VV1 and avelumab by the Investigator or Sponsor during the first Cycle (first 28 days) of study treatment except:  a. Diarrhea of ≤ 6 days duration following adequate and optimal therapy that resolves to Grade ≤ 1  b. Grade 3 skin toxicity that resolves to Grade 1 or less with supportive measures within 7 days  c. Nausea and vomiting of ≤ 72 h duration with adequate and optimal therapy  d. Single laboratory values out of the normal range that have no clinical correlate, and resolve to Grade ≤ 2 within 6 days with adequate medical management (except Grade ≥ 3 liver function test increase without any clinical correlate)  e. Grade 3 infusion-related reactions resolving within 6 h from the end of infusion and controlled with medical management  f. Transient (≤ 72 h) Grade 3 fatigue, local reactions, flu-like symptoms, fever, headache that resolves to Grade ≤ 1 with adequate treatment  g. Symptomatic thyroid dysfunction which is manageable with adequate treatment and resolves to Grade 2 within 6 days  h. Tumor flare phenomenon defined as local pain, irritation, or rash localized at sites of known or suspected tumor that resolve to Grade 2 within 6 days  i. Asymptomatic Grade ≥ 3 lipase or amylase elevation not associated with clinical manifestations of pancreatitis. |
| Any TEAE observed in subsequent cycle that in the opinion of the SMC needs to be taken into account for the overall evaluation of the safety and proposal of dose escalation | • Any Grade 4 neutropenia of > 5 days duration, Grade ≥ 3 febrile neutropenia.  • Grade 3 hemoglobin decrease (< 8.0 g/dL) despite blood transfusion or erythroid growth factor.  • Grade 4 hemoglobin decrease assessed as related to study drug.  • Any Grade 4 thrombocytopenia or Grade 3 thrombocytopenia with bleeding.  • Any Grade ≥ 3 clinical signs and symptoms related to increased QTc.  • Evidence of hepatocellular toxicity attributable to study drug, such as increase in AST or ALT ≥ 5-fold ULN for at least 72 hours or ≥ 3 x ULN elevation of ALT or AST and elevation of serum total bilirubin to ≥ 2 x ULN, without initial findings of cholestasis (elevated serum ALP) or other apparent clinical causality (e.g., viral hepatitis A, B, C, or co-medication). |
| Life-threatening VSV infection | • Grade 4 VSV infection lasting 6 days or more. |
| Increased viremia | • >10-fold increase in the copy number (copies/μg RNA) between sequential samples at least 3 days apart beyond day 14. |
