# Supplementary material for: Phase I Study of Oncolytic Virus VV1 as Monotherapy or in Combination with Avelumab in Patients with Relapsed Refractory Solid Tumors
Source: Cancer Res Commun. 2026 Aug 5;6(8):1862–74. doi: 10.1158/2767-9764.CRC-25-0803 (PMC13440224; doi:10.1158/2767-9764.CRC-25-0803)
Supplement: Supplementary file 8 [file crc-25-0803_table_s4_suppst4.docx]

**Table S4: Part 1 baseline demographics**

| **Dose** | **DL1**  **3 x 10^6^** | **DL2**  **1 x 10^7^** | **DL3**  **3 x 10^7^** | **DL4**  **1 x 10^8^** | **DL5**  **3 x 10^8^** | **DL6**  **1 x 10^9^** | **DL7**  **3 x 10^9^** | **Total** |
| --- | --- | --- | --- | --- | --- | --- | --- | --- |
|  | (N=3) | (N=3) | (N=4) | (N=4) | (N=4) | (N=6) | (N=3) | (N=27) |
| **Age (years)** | | | | | | | | |
| Median (min-max) | 57 (40-70) | 65 (59-79) | 62 (36-76) | 60 (38-73) | 65 (57-70) | 57 (47-74) | 61 (61-64) | 61 (36-79) |
| **Sex, n (%)** | | | | | | | | |
| Male | 2 (66.7) | 2 (66.7) | 2 (50.0) | 3 (75.0) | 3 (75.0) | 0 | 2 (66.7) | 14 (51.9) |
| Female | 1 (33.3) | 1 (33.3) | 2 (50.0) | 1 (25.0) | 1 (25.0) | 6 (100.0) | 1 (33.3) | 13 (48.1) |
| **Race** | | | | | | | | |
| Black or African American | 0 | 0 | 1 (25.0) | 0 | 1 (25.0) | 1 (16.7) | 0 | 3 (11.1) |
| White | 3 (100.0) | 3 (100.0) | 3 (75.0) | 4 (100.0) | 2 (50.0) | 5 (83.3) | 2 (66.7) | 22 (81.5) |
| Other | 0 | 0 | 0 | 0 | 0 | 0 | 1 (33.3) | 1 (3.7) |
| Not Reported | 0 | 0 | 0 | 0 | 1 (25.0) | 0 | 0 | 1 (3.7) |
| **Ethnicity** | | | | | | | | |
| Hispanic or Latino | 0 | 0 | 1 (25.0) | 1 (25.0) | 0 | 0 | 1 (33.3) | 3 (11.1) |
| Non-Hispanic or Latino | 3 (100.0) | 3 (100.0) | 3 (75.0) | 3 (75.0) | 4 (100.0) | 6 (100.0) | 2 (66.7) | 24 (88.9) |
| **Prior anti-cancer regimens** | | | | | | | | |
| Median (min–max) | 6.0 (6-7) | 3.0 (3–7) | 5.0 (4–5) | 4.0 (1–6) | 3.5 (2-4) | 4.5 (1-9) | 3.0 (1-6) | 4.0 (1-9) |
| **ECOG, n (%)** | | | | | | | | |
| Grade 0 | 2 (66.7) | 0 | 1 (25.0) | 0 | 3 (75.0) | 1 (16.7) | 1 (33.3) | 8 (29.6) |
| Grade 1 | 1 (33.3) | 3 (100.0) | 3 (75.0) | 4 (100.0) | 1 (25.0) | 5 (83.3) | 2 (66.7) | 19 (70.4) |
| **Child-Pugh Class, n (%)** | | | | | | | | |
| A (5-6 points) | 3 (100.0) | 2 (66.7) | 4 (100.0) | 4 (100.0) | 4 (100.0) | 6 (100.0) | 3 (100.0) | 26 (96.3) |
| Missing | 0 | 1 (33.3) | 0 | 0 | 0 | 0 | 0 | 1 (3.7) |

DL = dose level.
