# Supplementary material for: Phase I Study of Oncolytic Virus VV1 as Monotherapy or in Combination with Avelumab in Patients with Relapsed Refractory Solid Tumors
Source: Cancer Res Commun. 2026 Aug 5;6(8):1862–74. doi: 10.1158/2767-9764.CRC-25-0803 (PMC13440224; doi:10.1158/2767-9764.CRC-25-0803)
Supplement: Supplementary file 9 [file crc-25-0803_table_s5_suppst5.docx]

**Table S5: Part 2 baseline demographics**

| **Dose and IV Duration** | **1.7 x 10^10^**  **15 min [1]** | **1.7 x 10^10^**  **30 min** | **1.7 x 10^10^**  **60 min** | **1.7 x 10^10^**  **180 min** | **1.0 x 10^11^**  **30-90 min** | **1.7 x 10^10^**  **30 min^a^** | **Total** |
| --- | --- | --- | --- | --- | --- | --- | --- |
|  | (N=5) | (N=7) | (N=5) | (N=6) | (N=6) | (N=4) | (N=33) |
| **Age (years)** | | | | | | | |
| Median (min-max) | 64 (46-82) | 49 (19-70) | 53 (41-68) | 52 (37-67) | 60 (41-74) | 71 (66-74) | 61 (19-82) |
| **Sex, n (%)** | | | | | | | |
| Male | 1 (20.0) | 2 (28.6) | 2 (40.0) | 2 (33.3) | 3 (50.0) | 1 (25.0) | 11 (33.3) |
| Female | 4 (80.0) | 5 (71.4) | 3 (60.0) | 4 (66.7) | 3 (50.0) | 3 (75.0) | 22 (66.7) |
| **Race** | | | | | | | |
| Black or African American | 0 | 0 | 0 | 0 | 0 | 3 (75.0) | 3 (9.1) |
| White | 5 (100.0) | 7 (100.0) | 5 (100.0) | 6 (100.0) | 6 (100.0) | 1 (25.0) | 30 (90.9) |
| **Ethnicity** | | | | | | | |
| Hispanic or Latino | 1 (20.0) | 2 (28.6) | 2 (40.0) | 0 | 0 | 0 | 5 (15.2) |
| Non-Hispanic or Latino | 4 (80.0) | 5 (71.4) | 3 (60.0) | 6 (100.0) | 6 (100.0) | 4 (100.0) | 28 (84.8) |
| Unwilling to Provide | 0 | 0 | 0 | 0 | 0 | 0 | 0 |
| **Prior anti-cancer regimens^b^** | | | | | | | |
| Median (min-max) | 4.0 (2-9) | 3.0 (2-4) | 4.0 (2-7) | 3.5 (1-12) | 3.0 (1-6) | 2.0 (1-4) | 3.0 (1-12) |
| **ECOG, n (%)** | | | | | | | |
| Grade 0 | 3 (60.0) | 4 (57.1) | 4 (80.0) | 3 (50.0) | 3 (50.0) | 2 (50.0) | 19 (57.6) |
| Grade 1 | 2 (40.0) | 3 (42.9) | 1 (20.0) | 3 (50.0) | 3 (50.0) | 2 (50.0) | 14 (42.4) |
| **Child-Pugh Class, n (%)** | | | | | | | |
| A (5-6 points) | 5 (100.0) | 7 (100.0) | 5 (100.0) | 6 (100.0) | 6 (100.0) | 4 (100.0) | 33 (100.0) |

[1] 1.7 x 10^10^ TCID_50_ 15 min two DLTs occurred in five patients.

a = Expansion cohort, patients with NET.
b = Patients reporting.
