# Supplementary material for: Phase I Study of Oncolytic Virus VV1 as Monotherapy or in Combination with Avelumab in Patients with Relapsed Refractory Solid Tumors
Source: Cancer Res Commun. 2026 Aug 5;6(8):1862–74. doi: 10.1158/2767-9764.CRC-25-0803 (PMC13440224; doi:10.1158/2767-9764.CRC-25-0803)
Supplement: Supplementary file 10 [file crc-25-0803_table_s6_suppst6.docx]

**Table S6: Part 3 baseline demographics**

|  | **Safety Lead In**  **VV1 1.7 x 10^10^ +avelumab** | **RP2D  VV1 1.7 x 10^10^**  **+ avelumab (mCRC)** | **Total** |
| --- | --- | --- | --- |
|  | (N=3) | (N=13) | (N=16) |
| **Age (years)** | | | |
| Median (min-max) | 61 (57-64) | 59 (45-68) | 60 (45-68) |
| **Sex, n (%)** | | | |
| Male | 2 (66.7) | 9 (69.2) | 11 (68.8) |
| Female | 1 (33.3) | 4 (30.8) | 5 (31.3) |
| **Race** | | | |
| Black or African American | 0 | 1 (7.7) | 1 (6.3) |
| White | 3 (100) | 10 (76.9) | 13 (81.3) |
| Other/not reported | 0 | 2 (15.4) | 2 (12.5) |
| **Ethnicity** | | | |
| Hispanic or Latino | 1 (33.3) | 1 (7.7) | 2 (12.5) |
| Non-Hispanic or Latino | 2 (66.7) | 11 (84.6) | 13 (81.3) |
| Unwilling to Provide | 0 | 1 (7.7) | 1 (6.3) |
| **Prior anti-cancer regimens^a^** | | | |
| Median (min-max) | 4.0 (3-12) | 3.0 (2-6) | 3.0 (2-12) |
| **ECOG, n (%)** |  | | |
| Grade 0 | 2 (66.7) | 6 (46.2) | 8 (50.0) |
| Grade 1 | 1 (33.3) | 7 (53.8) | 8 (50.0) |
| **Child-Pugh Class, n (%)** | | | |
| A (5-6 points) | 3 (100) | 11 (84.6) | 14 (87.5) |
| B (7-9 points) | 0 | 2 (15.4) | 2 (12.5) |

Note: Percentages are based on the number of patients reporting non-missing results.
 a = Patients reporting.
