# Supplementary material for: Phase I Study of Oncolytic Virus VV1 as Monotherapy or in Combination with Avelumab in Patients with Relapsed Refractory Solid Tumors
Source: Cancer Res Commun. 2026 Aug 5;6(8):1862–74. doi: 10.1158/2767-9764.CRC-25-0803 (PMC13440224; doi:10.1158/2767-9764.CRC-25-0803)
Supplement: Supplementary file 11 [file crc-25-0803_table_s9_suppst9.docx]

**Table S9: VV1 neutralizing antibodies**

| **IT Dose**  **(TCID_50_)** | **Patient no** | **VV1 neutralizing antibody (μg of total RNA)** | | |
| --- | --- | --- | --- | --- |
|  |  | **Day 0** | **Day 29** | **Mean day 29** |
| 3 x 10^6^ | 1 | 0 | 640 | 253.3 |
|  | 2 | 0 | 80 |  |
|  | 3 | 0 | 40 |  |
| 1 x 10^7^ | 4 | 0 | 320* | 133.3 |
|  | 5 | 0 | 80 |  |
|  | 6 | 0 | 0 |  |
| 3 x 10^7^ | 7 | 0 | 0 | 725.0 |
|  | 8 | 0 | 20 |  |
|  | 9 | 0 | 2560 |  |
|  | 10 | 0 | 320 |  |
| 1 x 10^8^ | 11 | 0 | 320 | 175.0 |
|  | 12 | 0 | 20 |  |
|  | 13 | 0 | 40 |  |
|  | 14 | 0 | 320 |  |
| 3 x 10^8^ | 15 | 0 | 640 | 280.0 |
|  | 16 | 0 | 320 |  |
|  | 17 | 0 | 80 |  |
|  | 18 | 0 | 80 |  |
| 1 x 10^9^ | 19 | N/A | N/A | 168.0 |
|  | 20 | 0 | 40 |  |
|  | 21 | 0 | 80 |  |
|  | 22 | 0 | 320 |  |
|  | 23 | 0 | 80 |  |
|  | 24 | 0 | 320 |  |
| 3 x 10^9^ | 25 | 0 | 80 | 560.0 |
|  | 26 | 0 | 320 |  |
|  | 27 | 0 | 1280 |  |
| **IV (30 min)**  1.7 x 10^10^ | 28 | 0 | 1280** | 646.7 |
|  | 29 | 0 | 20** |  |
|  | 30 | 0 | 640** |  |

* Day 15 assessment. **Day 22 assessment.
